# Itaconate and fumarate derivatives inhibit priming and activation of the canonical NLRP3 inflammasome in macrophages

**DOI:** 10.1111/imm.13454

**Published:** 2022-03-02

**Authors:** Christopher Hoyle, Jack P. Green, Stuart M. Allan, David Brough, Eloise Lemarchand

**Affiliations:** ^1^ Geoffrey Jefferson Brain Research Centre The Manchester Academic Health Science Centre Northern Care Alliance NHS Group University of Manchester Manchester UK; ^2^ Division of Neuroscience and Experimental Psychology School of Biological Sciences Faculty of Biology, Medicine and Health University of Manchester Manchester UK; ^3^ The Lydia Becker Institute of Immunology and Inflammation University of Manchester Manchester UK; ^4^ INSERM UMR‐S U1237 Physiopathology and Imaging of Neurological Disorders Institut Blood and Brain @ Caen‐Normandie (BB@C) Normandie University Caen France

**Keywords:** fumarate, inflammasome, interleukin, itaconate, NLRP3

## Abstract

The NLRP3 inflammasome is a multiprotein complex that regulates caspase‐1 activation and subsequent interleukin (IL)‐1β and IL‐18 release from innate immune cells in response to infection or injury. Derivatives of the metabolites itaconate and fumarate, dimethyl itaconate (DMI), 4‐octyl itaconate (4OI) and dimethyl fumarate (DMF) limit both expression and release of IL‐1β following NLRP3 inflammasome activation. However, the direct effects of these metabolite derivatives on NLRP3 inflammasome responses require further investigation. Using murine bone marrow‐derived macrophages, mixed glia and organotypic hippocampal slice cultures (OHSCs), we demonstrate that DMI, 4OI and DMF pretreatments inhibit pro‐inflammatory cytokine production in response to lipopolysaccharide (LPS), as well as inhibit subsequent NLRP3 inflammasome activation induced by nigericin. DMI, 4OI, DMF and monomethyl fumarate (MMF), another fumarate derivative, also directly inhibited biochemical markers of NLRP3 activation in LPS‐primed macrophages, mixed glia, OHSCs and human macrophages in response to nigericin and imiquimod, including ASC speck formation, caspase‐1 activation, gasdermin D cleavage and IL‐1β release. DMF, an approved treatment of multiple sclerosis, as well as DMI, 4OI and MMF, inhibited NLRP3 activation in macrophages in response to lysophosphatidylcholine, which is used to induce demyelination, suggesting a possible mechanism for DMF in multiple sclerosis through NLRP3 inhibition. The derivatives also reduced pro‐IL‐1α cleavage in response to the calcium ionophore ionomycin. Together, these findings reveal the immunometabolic regulation of both the priming and activation steps of NLRP3 activation in macrophages. Furthermore, we highlight itaconate and fumarate derivatives as potential therapeutic options in NLRP3‐ and IL‐1α‐driven diseases, including in the brain.

## Graphical abstract

The effects of itaconate and fumarate derivatives on canonical NLRP3 inflammasome activation in macrophages and microglia were investigated. Itaconate and fumarate derivatives inhibited both the priming and activation steps of canonical NLRP3 activation in response to nigericin, imiquimod and lysophosphatidylcholine stimulation. The derivatives also reduced interleukin‐1α cleavage in response to ionomycin.

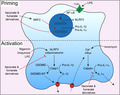

**Abbreviations**
4OI: 4‐octyl itaconate
ANOVA: analysis of variance
ASC: apoptosis‐associated speck‐like protein containing a caspase recruitment domain
BMDM: bone marrow‐derived macrophages
BSA: bovine serum albumin
DMEM: Dulbecco's modified Eagle's medium
DMF: dimethyl fumarate
DMI: dimethyl itaconate
ELISA: enzyme‐linked immunosorbent assay
FBS: fetal bovine serum
GSDMD‐NT: gasdermin D N‐terminal domain
IL: interleukin
KEAP1: Kelch‐like ECH‐associated protein 1
LDH: lactate dehydrogenase
LPC: lysophosphatidylcholine
LPS: lipopolysaccharide
M‐CSF: macrophage colony‐stimulating factor
MDM: monocyte‐derived macrophage
MMF: monomethyl fumarate
NLRP3: NLR family pyrin domain containing 3
NRF2: nuclear factor erythroid 2‐related factor 2
OHSC: organotypic hippocampal slice culture
PBMCs: peripheral blood mononuclear cells
PBS: phosphate‐buffered saline
PBST: PBS, 0.1% Tween‐20
PenStrep: 100 U ml^−1^ penicillin and 100 μg ml^−1^ streptomycin
PI: propidium iodide
SARS‐CoV‐2: severe acute respiratory syndrome coronavirus 2
SEM: standard error of the mean
TNF: tumour necrosis factor
WT: wild‐type

## INTRODUCTION

Macrophages are innate immune effector cells that regulate inflammatory responses upon infection or tissue injury to restore tissue homeostasis by promoting pathogen death or tissue and wound repair. In the brain, microglia are the resident macrophages and are important effectors of inflammatory responses. Inflammasomes are cytosolic complexes that regulate inflammatory responses in immune cells. In particular, the NLR family pyrin domain containing 3 (NLRP3) inflammasome is implicated in a range of non‐communicable diseases that are characterized by inflammation [1, 2]. Although several pathways of NLRP3 activation have been described [3, 4], canonical NLRP3 activation is the most studied. The canonical pathway consists of an initial priming step, typically mediated through Toll‐like receptor 4 (TLR4) signalling by bacterial endotoxin (or lipopolysaccharide, LPS) that upregulates NLRP3 and pro‐IL‐1β expression, followed by a subsequent NLRP3 activating stimulus. A broad range of pathogen‐ or damage‐associated molecular patterns (PAMPs and DAMPs, respectively) and environmental irritants are capable of providing this activating stimulus, and include the bacterial toxin and potassium ionophore nigericin, extracellular ATP [5], amyloid‐β aggregates [6] and silica crystals [7, 8]. The precise mechanism of NLRP3 activation is still unclear, with potassium efflux‐dependent [9] and efflux‐independent [10] mechanisms suggested to elicit disruption of subcellular organelle function, leading to inflammasome formation [11]. We recently summarized the evidence for organelle dysfunction as a crucial cellular event that leads to NLRP3 activation [12]. Once activated, NLRP3 interacts with the adaptor protein ASC (apoptosis‐associated speck‐like protein containing a caspase recruitment domain), causing the formation of an ASC speck that drives activation of the inflammasome effector protein caspase‐1 [13, 14]. Active caspase‐1 then cleaves gasdermin D, pro‐interleukin (IL)‐1β and pro‐IL‐18, with gasdermin D pores forming the conduit for mature IL‐1β release [15].

Immunometabolism has emerged as a regulator of macrophage inflammasome responses [16]. LPS treatment of macrophages causes a metabolic shift from oxidative phosphorylation to glycolysis that is necessary for IL‐1β production [17]. Certain metabolites such as itaconate [16], succinate [18] and fumarate [19] have immunoregulatory functions. For example, itaconate and fumarate derivatives, including dimethyl itaconate (DMI), 4‐octyl itaconate (4OI) and dimethyl fumarate (DMF), are able to activate nuclear factor erythroid 2‐related factor 2 (NRF2) signalling by alkylating and subsequently inducing the degradation of the cytoplasmic NRF2 inhibitor Kelch‐like ECH‐associated protein 1 (KEAP1) [20]. NRF2 is then able to translocate to the nucleus, where it not only upregulates the transcription of its target genes but also prevents the recruitment of RNA polymerase II to NF‐κB secondary response genes such as IL‐6 and IL‐1β [21]. DMI and 4OI also induce electrophilic stress and glutathione depletion in macrophages, which inhibits the LPS‐induced translation of IκBζ independently of NRF2, and this subsequently limits the expression of IκBζ‐dependent NF‐κB secondary response genes [22, 23]. NF‐κB primary response genes, such as TNF and NLRP3, are much less affected by NRF2 accumulation or IκBζ depletion [20, 22]. It must be acknowledged that the properties of these itaconate derivatives may not fully reflect the properties of endogenous itaconate [23]. In vivo evidence also indicates the importance of itaconate responses, as mice deficient in *Irg1*, which therefore cannot produce itaconate, rapidly succumb to *Mycobacterium tuberculosis* infection, whereas there is no mortality in wild‐type control mice [24]. Interestingly, 4OI and DMF exhibit antiviral and anti‐inflammatory effects through NRF2 signalling in response to severe acute respiratory syndrome coronavirus 2 (SARS‐CoV‐2) infection [25].

Although itaconate derivatives are known to limit pro‐IL‐1β expression, the direct effects of itaconate‐related molecules on NLRP3 inflammasome activation are less characterized. Previous studies suggest that DMF, an approved treatment of relapsing–remitting multiple sclerosis, and its metabolite monomethyl fumarate (MMF) limit NLRP3 inflammasome activation, with DMF exhibiting greater potency [26, 27]. DMF has also been shown to directly succinate a cysteine residue on gasdermin D to limit pyroptotic cell death in response to NLRP3 activation in vitro and in vivo, but without inhibiting NLRP3 activation itself [19]. Itaconate [23] and its derivative 4OI [28] can inhibit NLRP3 activation in an NRF2‐independent manner, with 4OI shown to modify specific cysteine residues on NLRP3, which may prevent NLRP3's interaction with NEK7 and subsequent activation [28]. IRG1‐deficient macrophages, which cannot synthesize endogenous itaconate, exhibit enhanced IL‐1β release in response to NLRP3 inflammasome activation [28, 29]. Finally, 4OI is effective at inhibiting NLRP3 activation in vivo [28].

Despite recent advances, further characterization of the effects of itaconate‐ and fumarate‐related compounds on NLRP3 inflammasome activation is required in order to evaluate their therapeutic potential. The relevance of immunometabolic regulation of microglial inflammasome responses in the brain is also unclear. Here, we demonstrate that itaconate and fumarate derivative pretreatment not only prevented expression of pro‐IL‐1β but also inhibited canonical NLRP3 inflammasome activation. We identified that itaconate and fumarate derivatives were able to directly inhibit canonical NLRP3 inflammasome activation, independent of their inhibitory effect on priming. These effects were consistent in mixed glia and organotypic hippocampal slice cultures (OHSCs), two brain‐relevant NLRP3 inflammasome models [30]. Itaconate and fumarate derivatives also inhibited NLRP3 activation induced by lysophosphatidylcholine (LPC), a lipid molecule used to induce demyelination in models of multiple sclerosis [31, 32, 33], further highlighting a potential mechanism of DMF action in multiple sclerosis treatment. The derivatives also inhibited pro‐IL‐1α cleavage following treatment of LPS‐primed BMDMs with the calcium ionophore ionomycin. These findings reveal multiple anti‐inflammatory effects of itaconate and fumarate derivatives in the innate immune system, through regulation of both the priming and activation steps of canonical NLRP3 inflammasome responses, and of the IL‐1α activation pathway.

## MATERIALS AND METHODS

### Mice

In‐house colonies of wild‐type (WT) and ASC–citrine [34] C57BL/6 mice at the University of Manchester were maintained to provide primary cell cultures. Animals were allowed free access to food and water and maintained under temperature‐, humidity‐ and light‐controlled conditions. All animal procedures adhered to the UK Animals (Scientific Procedures) Act (1986).

### Primary murine BMDM preparation

Primary bone marrow‐derived macrophages (BMDMs) were prepared by centrifuging the femurs of 3‐ to 6‐month‐old WT or ASC–citrine mice of either sex in an Eppendorf tube containing phosphate‐buffered saline (PBS) at 10 000 ***g*** (10 s). Bone marrow was collected, and red blood cells were lysed with ACK lysing buffer (Lonza, LZ10‐548E). Cells were passed through a cell strainer (70 µm pore size; Corning, 734‐2761) and centrifuged at 1500 ***g*** (5 min), and BMDMs were generated by resuspending and culturing the cell pellet in 70% Dulbecco's modified Eagle's medium (DMEM; Sigma, D6429) containing 10% (v/v) fetal bovine serum (FBS; Thermo, 10500064), 100 U ml^−1^ penicillin and 100 μg ml^−1^ streptomycin (PenStrep; Thermo, 15070063), and supplemented with 30% L929 mouse fibroblast‐conditioned medium for 7 days. Cells were incubated at 37°C, 90% humidity and 5% CO_2_. Before experiments, BMDMs were seeded overnight at a density of 1 × 10^6^ cells ml^−1^.

### Human THP‐1 preparation

THP‐1 cells were cultured in RPMI 1640 (Sigma, R8758) containing 10% (v/v) FBS and PenStrep at 37°C, 90% humidity and 5% CO_2_. Before experiments, THP‐1 cells were seeded at a density of 1 × 10^6^ cells ml^−1^ in culture medium containing phorbol 12‐myristate 13‐acetate (PMA; 500 nM) for 3 h to promote differentiation. Fresh culture medium was then applied, and cells were left overnight.

### Human monocyte‐derived macrophage preparation

Human monocyte‐derived macrophages (MDMs) were prepared from human peripheral blood mononuclear cells (PBMCs) obtained from consenting healthy donors (National Health Service Blood and Transplant, Manchester, UK), with full ethical approval from the University Research Ethics Committee at the University of Manchester (ref 2017‐2551‐3945). In brief, PBMCs were isolated by Ficoll separation (Thermo) at 400 ***g*** (40 min, room temperature) with zero deceleration. PBMCs were washed three times with sterile MACS buffer (0.5% (w/v) bovine serum albumin (BSA), 2 mM EDTA in PBS) before positive selection of CD14^+^ monocytes by incubation with magnetic CD14 microbeads (Miltenyi Biotec, 130‐050‐201) (15 min, 4°C) and elution using LS columns (Miltenyi Biotec, 130‐042‐401). CD14^+^ monocytes were differentiated to MDMs by culturing for 7 days (at a concentration of 1 × 10^6^ cells ml^−1^) in RPMI 1640 supplemented with 10% (v/v) FBS, PenStrep and macrophage colony‐stimulating factor (M‐CSF, 0.5 ng ml^−1^; PeproTech, 300‐25) at 37°C, 90% humidity and 5% CO_2_. On Day 3 of differentiation, cells were fed by the addition of fresh media containing M‐CSF (0.5 ng ml^−1^). Before experiments, MDMs were seeded overnight at a density of 1 × 10^6^ cells ml^−1^.

### Primary murine mixed glial culture preparation

Murine mixed glial cells were prepared from the brains of 2‐ to 4‐day‐old mice of either sex that were culled by cervical dislocation, as described previously [30]. The brains were isolated, cerebral hemispheres dissected and the meninges removed. The remaining brain tissue was homogenized in DMEM containing 10% (v/v) FBS and PenStrep via repeated trituration, then centrifuged at 500 ***g*** for 10 min, and the pellet was resuspended in fresh culture medium before being incubated in a flask at 37°C, 90% humidity and 5% CO_2_. After 5 days, the cells were washed, and fresh medium was applied. The medium was subsequently replaced every 2 days. On Day 12 of the culture, the cells were seeded at 2 × 10^5^ cells ml^−1^ in 24‐ or 48‐well plates and incubated for a further 2 days prior to use.

### Organotypic hippocampal slice culture preparation

Seven‐day‐old mouse pups of either sex were culled by cervical dislocation, and the brains were collected in PBS containing glucose (5 mg ml^−1^). The hippocampi were dissected and placed on filter paper, and 400‐μm slices were prepared using a McIlwain tissue chopper (Brinkman Instruments). Hippocampal slices were collected and placed on 0.4‐μm Millicell culture inserts (Merck Millipore, PICM03050), as described previously by Stoppini et al. (1991) [35]. Three hippocampal slices were placed on each insert. Slices were maintained in a humidified incubator with 5% CO_2_ at 37°C with 1 ml MEM (Gibco, 31095209) containing 20% (v/v) horse serum (Sigma, H1138), supplemented with HEPES (30 mM; Fisher, 10397023) and insulin (0.1 mg ml^−1^; Gibco, 12585014), pH 7.2–7.3. The culture medium was changed every 2 days, and slices were used at Day 7.

### Treatment protocols

To assess the effect of itaconate and fumarate derivative pretreatments on inflammasome priming, cells were first treated with vehicle (DMSO), DMI (125 µM, Sigma, 592498) or 4OI (125 µM; Cayman Chemical, CAY25374) for 20 h (BMDM) or 21 h (mixed glia and OHSC). Alternatively, BMDMs or mixed glia were treated with DMF (15 or 30 µM; Sigma, 242926) for 1 h. LPS (1 µg ml^−1^; Sigma, L2654) was then added to the wells for 4 h (BMDM) or 3 h (mixed glia and OHSC) to induce priming, followed by nigericin (10 µM; Sigma, N7143) for 60 min (BMDM and mixed glia) or 90 min (OHSC) to activate the NLRP3 inflammasome.

To assess the direct effect of itaconate and fumarate derivative treatments on canonical NLRP3 inflammasome activation in macrophages, BMDMs, human MDMs and THP‐1 cells were first primed with LPS (1 µg ml^−1^) for 4 h. The medium was then replaced with serum‐free DMEM (BMDM) or RPMI (human MDM, THP‐1) containing vehicle (DMSO), DMI, 4OI (125 µM, 15 min), DMF (30 or 125 µM, 15 min), MMF (500 µM, 15 min; Sigma, 651419), unmodified itaconate (1–7.5 mM, 15 min; Sigma, I29204) or the NLRP3 inhibitor MCC950 (10 µM, 15 min; Sigma, PZ0280), before nigericin (10 µM, 1, 4, or 20 h), imiquimod (75 µM, 2 h) or LPC (100 µM in ethanol, 60 min; Sigma, L4129) was added to the culture medium. To assess the direct effect of itaconate and fumarate derivative treatments on NLRP3 inflammasome activation in mixed glia and OHSCs, cells were first primed with LPS (1 µg ml^−1^) for 3 h. The medium was then replaced with serum‐free DMEM (mixed glia) or MEM (OHSC) containing vehicle (DMSO), DMI, 4OI, DMF (125 µM, 15 min) or MMF (500 µM, 15 min), before nigericin (10 µM, 1 h (mixed glia) or 1.5 h (OHSC)), imiquimod (75 µM, 2 h) or LPC (100 µM, 4 h) was added to the culture medium. To assess the effects of the derivatives on pro‐IL‐1α cleavage, BMDMs were primed with LPS (1 µg ml^−1^) for 4 h. The medium was then replaced with serum‐free DMEM containing vehicle (DMSO), DMI, 4OI (125 µM, 15 min or 3 h), DMF (30 or 125 µM, 15 min or 3 h), MMF (500 µM, 15 min or 3 h), necrostatin‐1 (50 µM, 15 min) or calpeptin (40 µM, 15 min or 3 h), before the addition of ionomycin (10 µM, 1 h; Cayman Chemical, CAY10004974) or ZVAD‐FMK (50 µM, 5 h; Sigma, 627610). At the end of the experiments, the supernatants were collected and cell or OHSC lysates prepared for further analysis.

### Western blotting

Primary BMDMs, mixed glia and OHSCs were lysed with lysis buffer (50 mM Tris–HCl, 150 mM NaCl; Triton‐X‐100 1% v/v, pH 7.3) containing protease inhibitor cocktail (Merck Millipore, 539131). OHSCs were additionally lysed using repeated trituration and brief water bath sonication. Lysates were then centrifuged for 10 min at 12 000 ***g*** at 4°C. In experiments where cells were lysed in‐well to assess total protein content in combined cell lysate and supernatant, cells were lysed by adding protease inhibitor cocktail and Triton‐X‐100 1% (v/v) into the culture medium. In‐well lysates were concentrated by mixing with an equal volume of trichloroacetic acid (Fisher, 10391351) and centrifuged for 10 min at 18 000 ***g*** at 4°C. The supernatant was discarded, and the pellet was resuspended in acetone (100%) before centrifugation for 10 min at 18 000 ***g*** at 4°C. The supernatant was again removed and the pellet allowed to air‐dry, before resuspending in Laemmli buffer (2×). Samples were analysed for NRF2, pro‐IL‐1β, mature IL‐1β, pro‐IL‐1α, mature IL‐1α, NLRP3, pro‐caspase‐1, caspase‐1 p10 and gasdermin D. Equal amounts of protein from lysates or equal volumes of in‐well lysates or supernatants were loaded into the gel. Samples were run on SDS–polyacrylamide gels and transferred at 25 V onto nitrocellulose or PVDF membranes using a Trans‐Blot^®^ Turbo Transfer™ System (Bio‐Rad). The membranes were blocked in either 5% w/v milk or 2.5% BSA (Sigma, A3608) in PBS, 0.1% Tween‐20 (PBST) for 1 h at room temperature. The membranes were then washed with PBST and incubated at 4°C overnight with goat anti‐mouse IL‐1β (250 ng ml^−1^; R&D Systems, AF‐401‐NA), goat anti‐mouse IL‐1α (100 ng ml^−1^; R&D Systems, AF‐400‐NA), mouse anti‐mouse NLRP3 (1 µg ml^−1^; Adipogen, G‐20B‐0014‐C100), rabbit anti‐mouse caspase‐1 (1.87 µg ml^−1^; Abcam, ab179515), rabbit anti‐mouse gasdermin D (0.6 µg ml^−1^; Abcam, ab209845) or rabbit anti‐mouse NRF2 (1.5 µg ml^−1^; CST, 12721) primary antibodies in 0.1% (IL‐1β, IL‐1α), 1% (NLRP3) or 2.5% (caspase‐1, gasdermin D, NRF2) BSA in PBST. The membranes were washed and incubated with rabbit anti‐goat IgG (500 ng ml^−1^, 5% milk in PBST; Agilent, P044901‐2), rabbit anti‐mouse IgG (1.3 µg ml^−1^, 5% milk in PBST; Agilent, P026002‐2) or goat anti‐rabbit IgG (250 ng ml^−1^, 2.5% BSA in PBST; Agilent, P044801‐2) at room temperature for 1 h. Proteins were then visualized with Amersham ECL Western Blotting Detection Reagent (GE Healthcare, RPN2236) and G:BOX (Syngene) and Genesys software. β‐Actin (Sigma, A3854) was used as a loading control. Densitometry was performed using Fiji (ImageJ). Uncropped Western blots are provided in Figures S14–S20.

### ELISA

The levels of IL‐1β, IL‐6 and tumour necrosis factor (TNF) in the supernatant were analysed by enzyme‐linked immunosorbent assay (ELISA; DuoSet, R&D Systems) according to the manufacturer's instructions.

### Cell death assays

Cell death was assessed by measuring lactate dehydrogenase (LDH) release into the supernatant using a CytoTox 96 Non‐Radioactive Cytotoxicity Assay (Promega) according to the manufacturer's instructions, and expressed relative to total cell death. Cell death in OHSCs was assessed by adding propidium iodide (25 µg ml^−1^; Sigma, P4864) to the culture medium for the final 30 min of the inflammasome activation protocol followed by widefield microscopy, or by relative LDH release when propidium iodide staining was not possible.

### Live imaging of ASC speck formation

ASC–citrine‐expressing primary BMDMs were used to perform live imaging of ASC speck formation. For itaconate derivative pretreatment assays, cells were seeded at 1 × 10^6^ cells ml^−1^ in black‐walled 96‐well plates and incubated for 1 h, and were then treated with vehicle (DMSO), DMI or 4OI (125 µM, 20 h). LPS (1 µg ml^−1^, 4 h) was then added to the wells to induce priming. In separate experiments, cells were seeded out overnight and treated with vehicle or DMF (15 or 31 µM, 1 h) the following day before LPS priming. The medium was replaced with OptiMEM, and nigericin (10 µM) was added to activate the NLRP3 inflammasome. For assays where itaconate derivative treatments were added after LPS priming, cells were seeded overnight at 1 × 10^6^ cells ml^−1^ in 96‐well plates. Cells were then first primed with LPS (1 µg ml^−1^, 4 h). The medium was replaced with OptiMEM containing vehicle, DMI, 4OI, DMF (125 µM), MMF (500 µM) or MCC950 (10 µM, 15 min) prior to addition of nigericin (10 µM) or LPC (100 µM). Image acquisition began immediately after nigericin treatment. Images were subsequently acquired every 10 min for a further 90 min using either an IncuCyte ZOOM^®^ or S3 Live Cell Analysis System (Essen Bioscience) at 37°C using a 20×/0.61 S Plan Fluor objective. Speck number was quantified manually and was assessed for each treatment at the final time‐point of 90 min.

### OHSC immunostaining

Organotypic hippocampal slice cultures were washed once with cold PBS and fixed in 4% paraformaldehyde (1 h) at 4°C. OHSCs were washed two more times in cold PBS and then incubated with rabbit anti‐mouse ASC (202 ng ml^−1^; CST, 67824) primary antibody overnight at 4°C. OHSCs were washed and incubated with Alexa Fluor™ 488 donkey anti‐rabbit IgG (2 µg ml^−1^; Invitrogen, A‐21206) secondary antibody for 2 h at room temperature. All antibody incubations were performed using PBS, 0.3% Triton‐X‐100. Wash steps were performed using PBST unless stated otherwise. OHSCs were washed and then incubated in DAPI (1 µg ml^−1^, 15 min; Sigma, D9542) at room temperature before final washing and mounting using ProLong™ Gold Antifade Mountant (Thermo, P36934) prior to imaging using widefield microscopy.

### Snapshot widefield microscopy

Images were collected on a Zeiss Axioimager.M2 upright microscope using a 5× or 20× Plan Apochromat objective and captured using a Coolsnap HQ2 camera (Photometrics) through Micromanager software (v1.4.23). Specific band‐pass filter sets for DAPI and FITC were used to prevent bleed‐through from one channel to the next.

### Image processing analysis

Analysis was performed using Fiji (ImageJ) on images acquired from the same region of up to three separate OHSCs (from the same insert) per treatment, and these values were averaged for each biological repeat. ASC speck formation was quantified on 20× widefield microscopy images by subtracting background (50 pixel rolling ball radius), manually setting thresholds and analysing particles with the following parameters: size 1–10 μm^2^, circularity 0.9–1.0. To quantify PI uptake, images were acquired on a widefield microscope using a 5× objective, background was subtracted (5.0 pixel rolling ball radius), and thresholds for images were automatically determined using the default method. The total area of PI‐positive signal was measured in the whole field of view and was then normalized to the total area of DAPI signal.

### Calpain activity assay

To assess the effect of DMF on calpain activity, WT BMDMs were primed with LPS (1 µg ml^−1^) for 4 h before treatment with vehicle (DMSO) or DMF (125 µM) for 1 h. Vehicle or ionomycin (10 µM) was then added to the well for 5 min. Calpain activity was assessed using the calpain activity assay kit (Abcam, ab65308) according to the manufacturer's instructions. Briefly, at the end of the ionomycin stimulation, the supernatant was then collected for LDH release and pro‐IL‐1α cleavage analysis, and the cell lysate was collected in extraction buffer prior to centrifugation at 18 000 ***g*** at 4°C for 5 min, after which the supernatant was incubated with reaction buffer and fluorescent substrate (Ac‐LLY‐AFC) for 1 h at 37°C, prior to measuring the fluorescence (400/505 nm).

### Data analysis

Data are presented as the mean ± standard error of the mean (SEM) together with individual data points where possible. Data were analysed using the unpaired t‐test, unmatched or repeated‐measures one‐way or two‐way analysis of variance (ANOVA), or mixed‐effects model, with Dunnett's or Sidak's post hoc test using GraphPad Prism (v8). Transformations or corrections were applied as necessary to obtain equal variance between groups prior to analysis. Statistical significance was accepted at *p < 0.05.

## RESULTS

### Pretreatment with itaconate and fumarate derivatives reduces priming and activation of the canonical NLRP3 inflammasome

**FIGURE 1:**
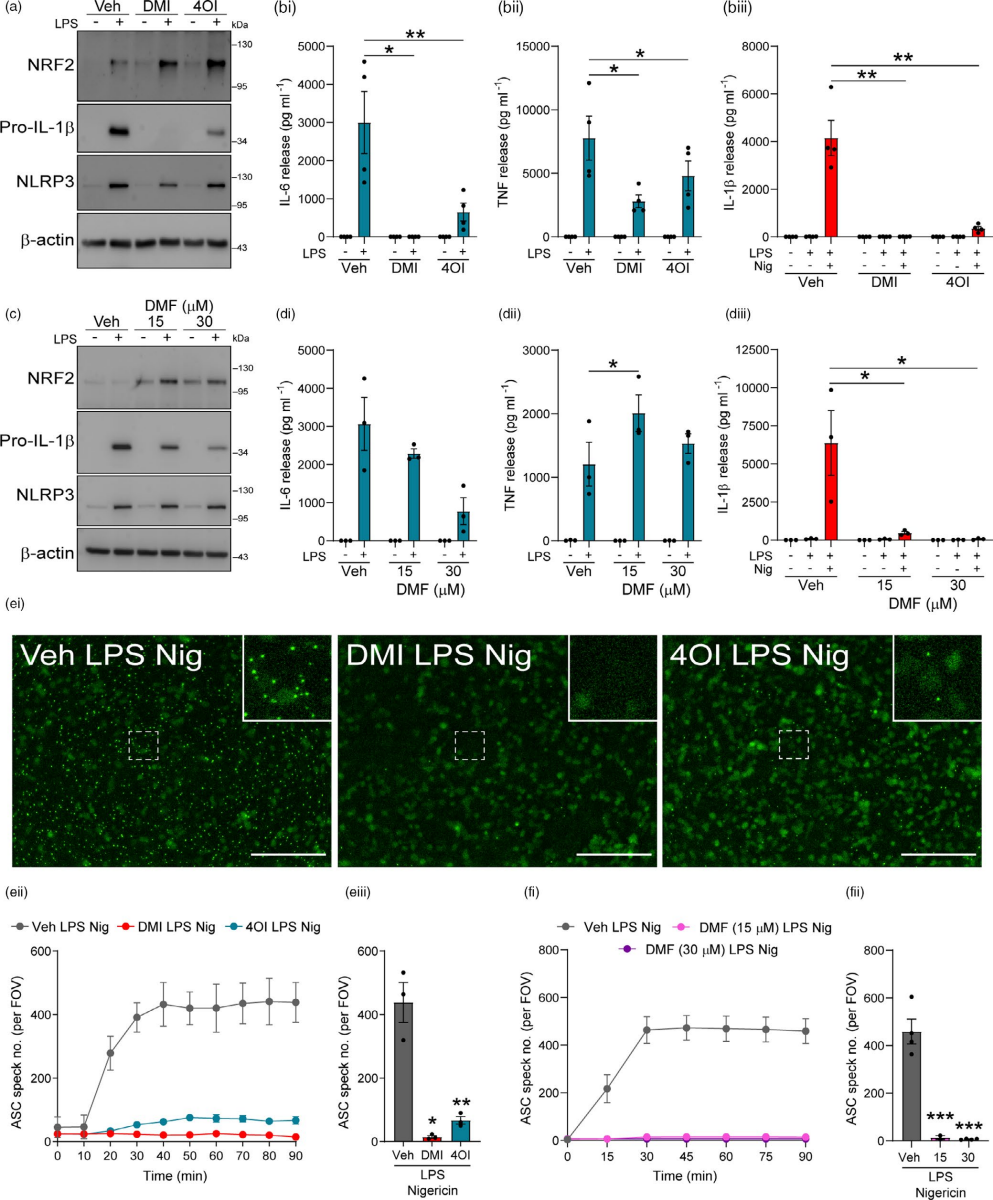
Pretreatment with itaconate and fumarate derivatives reduces priming and activation of the canonical NLRP3 inflammasome. (a) WT BMDMs were treated with vehicle (DMSO), DMI or 4OI (125 µM, 20 h). LPS (1 µg ml^−1^, 4 h) was then added to the wells to induce priming (*n* = 4). Cell lysates were probed by Western blotting for NRF2, pro‐IL‐1β and NLRP3 protein. For densitometry, see Figure S1A. (b) WT BMDMs were treated as above, followed by nigericin (10 µM, 60 min; *n* = 4). Supernatants were assessed for (bi) IL‐6, (bii) TNF and (biii) IL‐1β content by ELISA. (c) WT BMDMs were treated with vehicle (DMSO) or DMF (15 or 30 µM, 1 h), followed by LPS (1 µg ml^−1^, 4 h) to induce priming (*n* = 3). Cell lysates were probed by Western blotting for NRF2, pro‐IL‐1β and NLRP3 protein. For densitometry, see Figure S1D. (d) WT BMDMs were treated as above, followed by nigericin (10 µM, 60 min; *n* = 3). Supernatants were assessed for (di) IL‐6, (dii) TNF and (diii) IL‐1β content by ELISA. (e, f) ASC–citrine BMDMs were treated as in (a) and (c), followed by nigericin (10 µM, 90 min). ASC speck formation was measured over a period of 90 min. Image acquisition began immediately after addition of nigericin. (ei) Fluorescence images after 90‐min nigericin stimulation following Veh, DMI or 4OI treatment are shown. Scale bars are 200 µm. ASC speck number per field of view was measured over 90 min, and quantification at the final time‐point of 90 min is shown following (eii, iii) vehicle, DMI or 4OI (*n* = 3), or (fi, ii) vehicle or DMF (*n* = 2–4) treatment. Data are presented as mean ± SEM. Data were analysed using unmatched one‐way (fii), repeated‐measures one‐way (eiii) or repeated‐measures two‐way (bi–iii, di–iii) ANOVA with Dunnett's post hoc test (vs. Veh treatment within each group). *p < 0.05; **p < 0.01; and ***p < 0.001. BMDMs, bone marrow‐derived macrophages; DMF, dimethyl fumarate; DMI, dimethyl itaconate; LPS, lipopolysaccharide; WT, wild‐type

Bone marrow‐derived macrophages were pretreated with two cell‐permeable derivatives of itaconate, DMI and 4OI, as well as the fumarate derivative DMF, and the effects on NLRP3 priming and activation were assessed. DMI and 4OI treatment alone did not induce NRF2 accumulation in murine WT BMDMs, but both enhanced LPS‐induced NRF2 accumulation (Figure 1a, Figure S1Ai). DMI and 4OI pretreatment inhibited the production of pro‐IL‐1β in response to LPS priming, and whilst DMI reduced NLRP3 expression, 4OI had no effect on NLRP3 levels (Figure 1a, Figure S1Aii, Aiii). DMI and 4OI also strongly reduced LPS‐induced IL‐6 and TNF release (Figure 1bi, bii). Consistent with inhibition of pro‐IL‐1β expression, DMI and 4OI pretreatment blocked IL‐1β release in response to subsequent stimulation with LPS and nigericin (Figure 1biii). Although DMF was included in these experiments at the same dose, subsequent assessment of the toxicity of the compounds revealed that DMF was toxic at this dose and duration of treatment; hence, these data were not included in further analysis (Figure S1B). Instead, WT BMDMs were treated with lower doses of DMF for a shorter duration that were not toxic (Figure S1C). Non‐toxic doses of DMF alone induced NRF2 accumulation, which was enhanced by the addition of LPS (Figure 1c, Figure S1Di). DMF also reduced pro‐IL‐1β levels in response to LPS priming, but did not affect NLRP3 expression (Figure 1c, Figure S1Dii, Diii). DMF caused a modest (but non‐significant) reduction in IL‐6 release in response to LPS priming, but did not inhibit TNF release, and DMF also blocked IL‐1β release following subsequent LPS and nigericin stimulation (Figure 1di‐iii).

We next investigated whether itaconate and fumarate derivative pretreatment could reduce NLRP3 inflammasome activation, as has been recently suggested [23]. BMDMs from ASC–citrine reporter mice [34] were treated with DMI and 4OI prior to LPS priming and subsequent nigericin treatment. Representative images of ASC speck formation from this experiment after 90 min of nigericin treatment are shown (Figure 1ei). DMI and 4OI pretreatment inhibited the formation of ASC specks (Figure 1eii, eiii). ASC–citrine BMDMs were also treated with DMF prior to LPS priming and nigericin treatment, and DMF was also found to inhibit ASC speck formation (Figure 1fi, fii, Figure S2). These data suggested that itaconate and fumarate derivative pretreatment may additionally inhibit the NLRP3 inflammasome activation step, as well as inhibit the priming stage.

### Itaconate and fumarate derivatives directly inhibit the NLRP3 activation step

**FIGURE 2:**
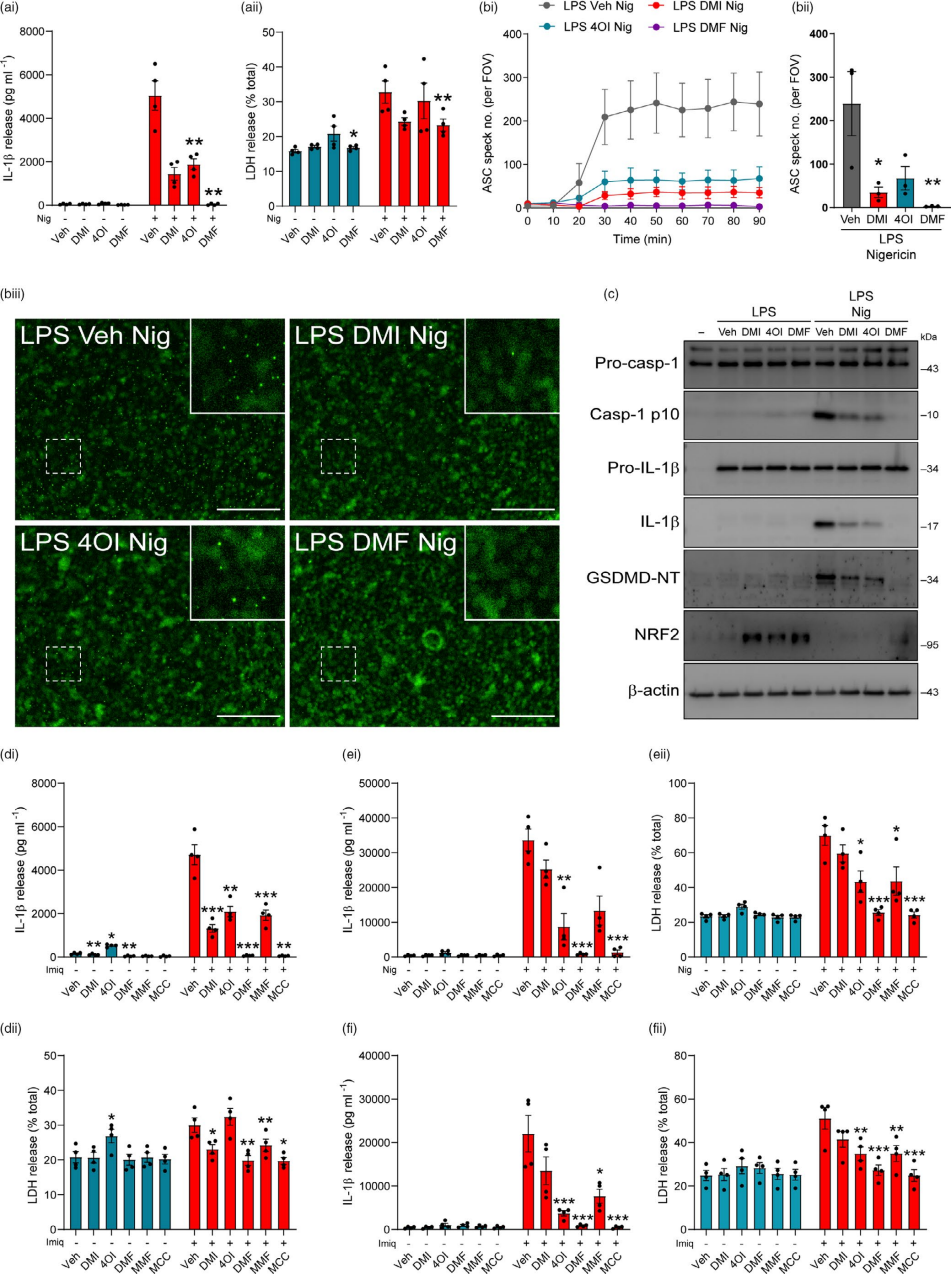
Itaconate and fumarate derivatives directly inhibit the NLRP3 activation step in murine and human macrophages. (a) WT BMDMs were primed with LPS (1 µg ml^−1^, 4 h) before treatment with vehicle (DMSO), DMI, 4OI or DMF (125 µM, 15 min). Nigericin was then added to the well (10 µM, 60 min; *n* = 4). Supernatants were assessed for (ai) IL‐1β release and (aii) cell death (LDH release). (b) ASC–citrine BMDMs were treated as above, and ASC speck formation was measured over a period of 90 min (*n* = 3). Image acquisition began immediately after addition of nigericin. (bi) ASC speck number per field of view was quantified over 90 min. (bii) ASC speck number and (biii) fluorescence images after 90‐min nigericin treatment are shown. Scale bars are 200 µm. (c) WT BMDMs were treated as above and then lysed in‐well, and combined cell lysate and supernatant was probed for several markers of inflammasome activation by Western blotting (*n* = 4) (GSDMD‐NT, gasdermin D N‐terminal domain). For densitometry, see Figure S3B. (d) WT BMDMs were LPS‐primed (1 µg ml^−1^, 4 h) before treatment with vehicle (DMSO), DMI, 4OI or DMF (125 µM, 15 min) or MMF (500 µM, 15 min). Imiquimod was then added to the well (75 µM, 2 h; *n* = 4). Supernatants were assessed for (di) IL‐1β release and (dii) LDH release. (e, f) PMA‐differentiated THP‐1 macrophages were primed with LPS (1 µg ml^−1^, 4 h) before treatment with vehicle, DMI, 4OI or DMF (125 µM), MMF (500 µM) or MCC950 (MCC, 10 µM, 15 min). (e) Nigericin was then added to the well (10 µM, 60 min; *n* = 4). Supernatants were assessed for (ei) IL‐1β release and (eii) LDH release. (f) Imiquimod was then added to the well (75 µM, 2 h; *n* = 4). Supernatants were assessed for (fi) IL‐1β release and (fii) LDH release. Supernatants were assessed for cytokine content by ELISA. Data are presented as mean ± SEM. Data were analysed using repeated‐measures one‐way (bii) or two‐way (a, d) ANOVA, or unmatched two‐way ANOVA (e, f), with Dunnett's post hoc test (vs. Veh treatment within each group). *p < 0.05; **p < 0.01; and ***p < 0.001. BMDMs, bone marrow‐derived macrophages; DMF, dimethyl fumarate; DMI, dimethyl itaconate; LDH, lactate dehydrogenase; LPS, lipopolysaccharide; MMF, monomethyl fumarate; WT, wild‐type

To determine whether NLRP3 inflammasome inhibition by itaconate derivatives was direct, LPS‐primed WT BMDMs were treated with DMI, 4OI and DMF prior to nigericin stimulation, and this resulted in inhibition of IL‐1β release, as well as small reductions in cell death (Figure 2ai, aii). Western blotting of the cell lysates demonstrated that the levels of pro‐IL‐1β were consistent between treatments, confirming that in this protocol expression of pro‐IL‐1β was unaffected by DMI, 4OI or DMF (Figure S3Ai, Aii). Dose‐dependent inhibition of IL‐1β release was observed for each treatment, with minimal reductions in cell death (Figure S4). To assess the effects of the derivatives on ASC speck formation, LPS‐primed ASC–citrine BMDMs were treated with DMI, 4OI and DMF prior to nigericin stimulation. Each of these metabolite derivatives inhibited ASC speck formation in response to nigericin, suggesting that these compounds were also able to directly block NLRP3 inflammasome activation independently of their effects on the priming response (Figure 2bi–iii). Fluorescence images of ASC speck formation are shown (Figure 2biii). LPS‐primed primary BMDMs treated with DMI, 4OI or DMF and subsequent nigericin stimulation were lysed directly in‐well without removing the supernatant, and western blotting of combined cell lysate and supernatant confirmed reductions in caspase‐1 activation, and gasdermin D and IL‐1β cleavage (Figure 2c, Figure S3B). NRF2 levels were increased by DMI, 4OI and DMF treatment after LPS priming, although this was not observed in cells that received subsequent nigericin stimulation (Figure 2c, Figure S3B), and itaconate‐mediated NLRP3 inhibition is suggested to be independent of NRF2 [23, 28]. MMF treatment limited NLRP3 activation in LPS‐primed BMDMs, although it was not as potent as DMF (Figure S5A). We confirmed that unmodified itaconate treatment inhibited NLRP3 activation in LPS‐primed BMDMs, although much higher doses were required because it is less cell‐permeable (Figure S5B) [23]. The itaconate and fumarate derivatives also inhibited potassium efflux‐independent NLRP3 activation in response to imiquimod stimulation (Figure 2di, dii). We also assessed whether the derivatives could still inhibit NLRP3 in response to prolonged nigericin stimulation. Whilst the derivatives maintained their inhibition of IL‐1β release following either 4 or 20 h of nigericin stimulation, at 4 h 4OI was no longer able to inhibit nigericin‐induced cell death (Figure S6A), and at 20 h, none of the derivatives, nor MCC950, a selective NLRP3 inhibitor [36], were able to inhibit nigericin‐induced cell death (Figure S6B). These data suggest that cell death after prolonged nigericin stimulation is independent of NLRP3 and the derivatives are unable to block it.

To determine whether the inhibitory effects of itaconate and fumarate derivatives were relevant in human macrophages, PMA‐differentiated THP‐1 macrophages, a human macrophage cell line, were LPS‐primed before treatment with DMI, 4OI and DMF and subsequent nigericin or imiquimod stimulation. The derivatives reduced IL‐1β release in response to both NLRP3 stimuli, accompanied by reductions in cell death, although DMI did not have a clear effect (Figure 2e, f). We confirmed these findings in LPS‐primed primary human macrophages, in which both DMI and DMF reduced nigericin‐induced IL‐1β release, whereas 4OI did not significantly reduce IL‐1β release at this dose (Figure S7A). Given the variability of IL‐1β release, likely due to donor variability, we have also expressed IL‐1β release relative to vehicle and nigericin treatment (Figure S7B). No inhibition of cell death was observed, although clear nigericin‐induced cell death was not always observed (Figure S7C). Thus, the itaconate and fumarate derivatives were able to directly inhibit NLRP3 activation in murine and human peripheral macrophages, independent of their effects on priming.

### Itaconate and fumarate derivative pretreatment inhibits priming and activation of the canonical NLRP3 inflammasome in mixed glia and OHSCs

**FIGURE 3:**
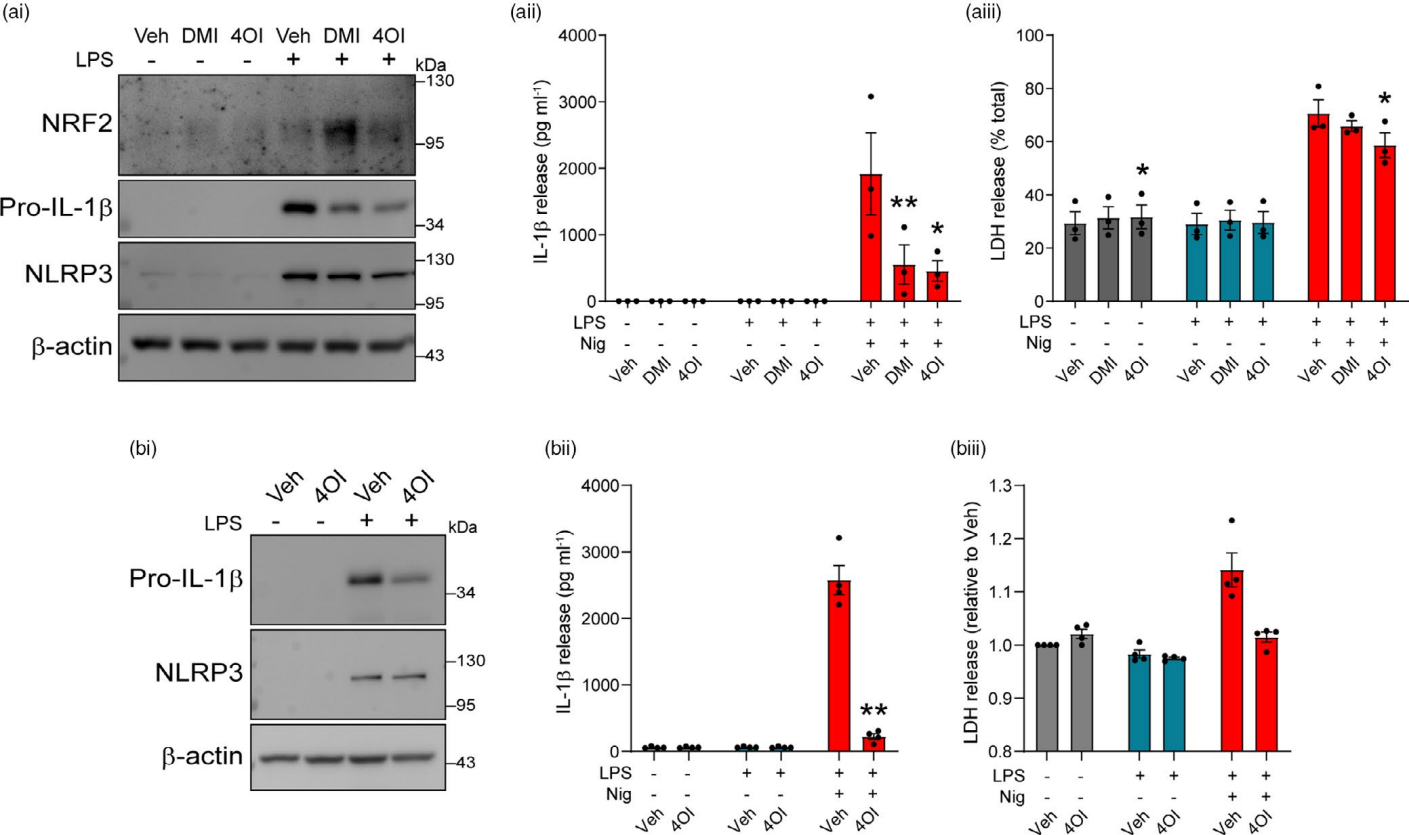
Itaconate and fumarate derivative pretreatment inhibits priming of the NLRP3 inflammasome in mixed glia and OHSCs. (a) WT mixed glia were treated with vehicle (DMSO), DMI or 4OI (125 µM, 21 h). LPS (1 µg ml^−1^, 3 h) was then added to the wells to induce priming (*n* = 3). (ai) Cell lysates were probed by Western blotting for NRF2, pro‐IL‐1β and NLRP3 protein. For densitometry, see Figure S8A. (aii, aiii) WT mixed glia were treated as above, followed by nigericin (10 µM, 60 min; *n* = 3). Supernatants were assessed for (aii) IL‐1β release and (aiii) cell death (LDH release). (b) WT OHSCs were treated with vehicle (DMSO) or 4OI (125 µM, 21 h). LPS (1 µg ml^−1^, 3 h) was then added to the wells to induce priming (*n* = 4). (bi) OHSC lysates were probed by Western blotting for pro‐IL‐1β and NLRP3 protein. For densitometry, see Figure S8C. (bii, biii) WT OHSCs were treated as above, followed by nigericin (10 µM, 90 min; *n* = 4). Supernatants were assessed for (bii) IL‐1β release and (biii) LDH release. Supernatants were assessed for cytokine content by ELISA. Data are presented as mean ± SEM. Data were analysed using repeated‐measures two‐way ANOVA with Dunnett's (a) or Sidak's (b) post hoc test (vs. Veh treatment within each group). *p < 0.05; **p < 0.01. BMDMs, bone marrow‐derived macrophages; DMF, dimethyl fumarate; DMI, dimethyl itaconate; LDH, lactate dehydrogenase; LPS, lipopolysaccharide; MMF, monomethyl fumarate; OHSC, organotypic hippocampal slice culture; WT, wild‐type

Despite accumulating evidence in peripheral immune cells, little is known about the importance of immunometabolic regulation of NLRP3 inflammasome priming and activation in the brain, where microglia are thought to be the predominant source of inflammasomes [37]. Thus, we assessed the effect of itaconate derivative pretreatment on mixed glial cultures, which consist of approximately 80% astrocytes, 10% microglia and 10% oligodendrocyte/type 2 astrocyte progenitor cells [38], and organotypic hippocampal slice cultures (OHSCs), which we recently validated as a model for studying microglial NLRP3 responses [30]. DMI and 4OI alone did not induce detectable NRF2 accumulation in mixed glial cultures (Figure 3ai, Figure S8Ai). DMI treatment did not significantly enhance LPS‐induced NRF2 accumulation, although increases were observed; similarly, 4OI did not increase LPS‐induced NRF2 levels (Figure 3ai, Figure S8Ai). Despite not enhancing NRF2 accumulation, both DMI and 4OI reduced LPS‐induced pro‐IL‐1β expression in mixed glial cultures, with marginal (and non‐significant) effects on NLRP3 protein levels (Figure 3ai, Figure S8Aii, Aiii). IL‐1β release upon subsequent stimulation with nigericin was also inhibited by DMI and 4OI, and 4OI caused a modest reduction in cell death (Figure 3aii, aiii). Following the same protocol as in the BMDMs to avoid toxicity, a shorter duration of DMF pretreatment induced NRF2 accumulation in the mixed glia, accompanied by apparent reductions in pro‐IL‐1β levels following LPS priming, albeit not statistically significant (Figure S8Bi‐iii). No reductions were observed in NLRP3 expression (Figure S8Bi, Biv). DMF has been previously demonstrated to inhibit IL‐1β, IL‐6 and to a lesser extent TNF expression at a lower dose in rat neonatal microglial cultures [39]. DMF pretreatment also reduced the release of IL‐1β following subsequent nigericin stimulation, although this was not statistically significant, and there was no inhibition of nigericin‐induced cell death (Figure S8Bv, Bvi). Given that the inhibition of pro‐IL‐1β production and mature IL‐1β release was comparable between DMI, 4OI and DMF in mixed glia, only 4OI was used in OHSCs, in which we have previously shown that IL‐1β production is almost exclusively a microglial response [30]. NRF2 accumulation could not be reliably detected in OHSCs upon LPS priming (data not shown). 4OI reduced the production of pro‐IL‐1β in response to LPS priming, but did not affect NLRP3 production (Figure 3bi, Figure S8Ci, Cii). 4OI pretreatment strongly inhibited IL‐1β release in response to LPS and nigericin treatment, although the reduction in cell death was not statistically significant (Figure 3bii, biii). These data suggested that itaconate derivatives were able to limit the priming of inflammasome responses and that this may be a relevant mechanism to regulate microglial inflammatory gene expression.

### Itaconate and fumarate derivatives directly inhibit activation of the canonical NLRP3 inflammasome in mixed glia and OHSCs

**FIGURE 4:**
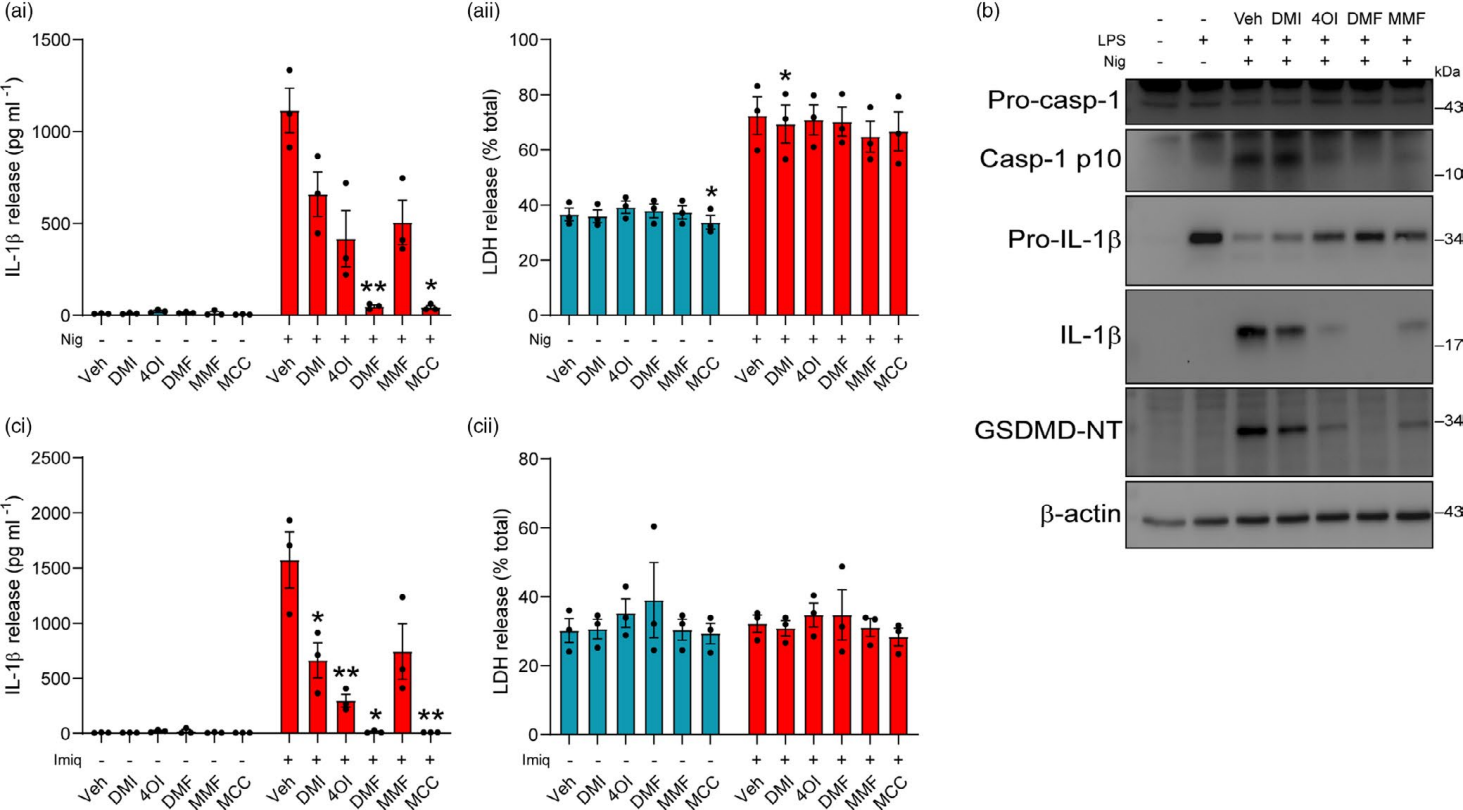
Itaconate and fumarate derivatives inhibit canonical NLRP3 activation in LPS‐primed mixed glia. Mixed glia were primed with LPS (1 µg ml^−1^, 3 h) before treatment with vehicle, DMI, 4OI, DMF (125 µM), MMF (500 µM) or MCC950 (MCC, 10 µM, 15 min). (a) Nigericin was then added to the well (10 µM, 60 min; *n* = 3), and supernatants were assessed for (ai) IL‐1β release and (aii) cell death (LDH release), or (b) cells were lysed in‐well, and combined cell lysate and supernatant was probed for several markers of inflammasome activation by Western blotting (*n* = 3) (GSDMD‐NT, gasdermin D N‐terminal domain). For densitometry, see Figure S9. (c) Mixed glia were treated as above, and imiquimod was then added to the well (75 µM, 2 h; *n* = 3). Supernatants were assessed for (ci) IL‐1β release and (cii) cell death (LDH release). Data are presented as mean ± SEM. Data were analysed using repeated‐measures two‐way ANOVA (a,c) with Dunnett's post hoc test (vs. Veh treatment within each group). *p < 0.05; **p < 0.01. DMF, dimethyl fumarate; DMI, dimethyl itaconate; LDH, lactate dehydrogenase; LPS, lipopolysaccharide; MMF, monomethyl fumarate; OHSC, organotypic hippocampal slice culture

We next investigated whether itaconate and fumarate derivatives could directly inhibit NLRP3 inflammasome activation in mixed glia and OHSCs. Previously, we have shown that MCC950 potently inhibits NLRP3 activation in both mixed glia and OHSCs, indicating that pharmacological inhibition of NLRP3 is possible in these in vitro microglial models [30]. LPS‐primed mixed glial cultures were treated with the derivatives prior to nigericin stimulation, and whilst DMI, 4OI and MMF caused modest but non‐significant reductions in IL‐1β release, DMF and MCC950 potently inhibited IL‐1β release but did not inhibit cell death (Figure 4ai, aii). LPS‐primed mixed glia were treated as above and then were lysed directly in‐well without removing the supernatant, and Western blotting of combined cell lysate and supernatant confirmed reductions in caspase‐1 activation, gasdermin D cleavage and IL‐1β processing (Figure 4b, Figure S9). The itaconate and fumarate derivatives also inhibited imiquimod‐induced IL‐1β release from LPS‐primed mixed glia, with no effect on cell death (Figure 4ci, cii).

**FIGURE 5:**
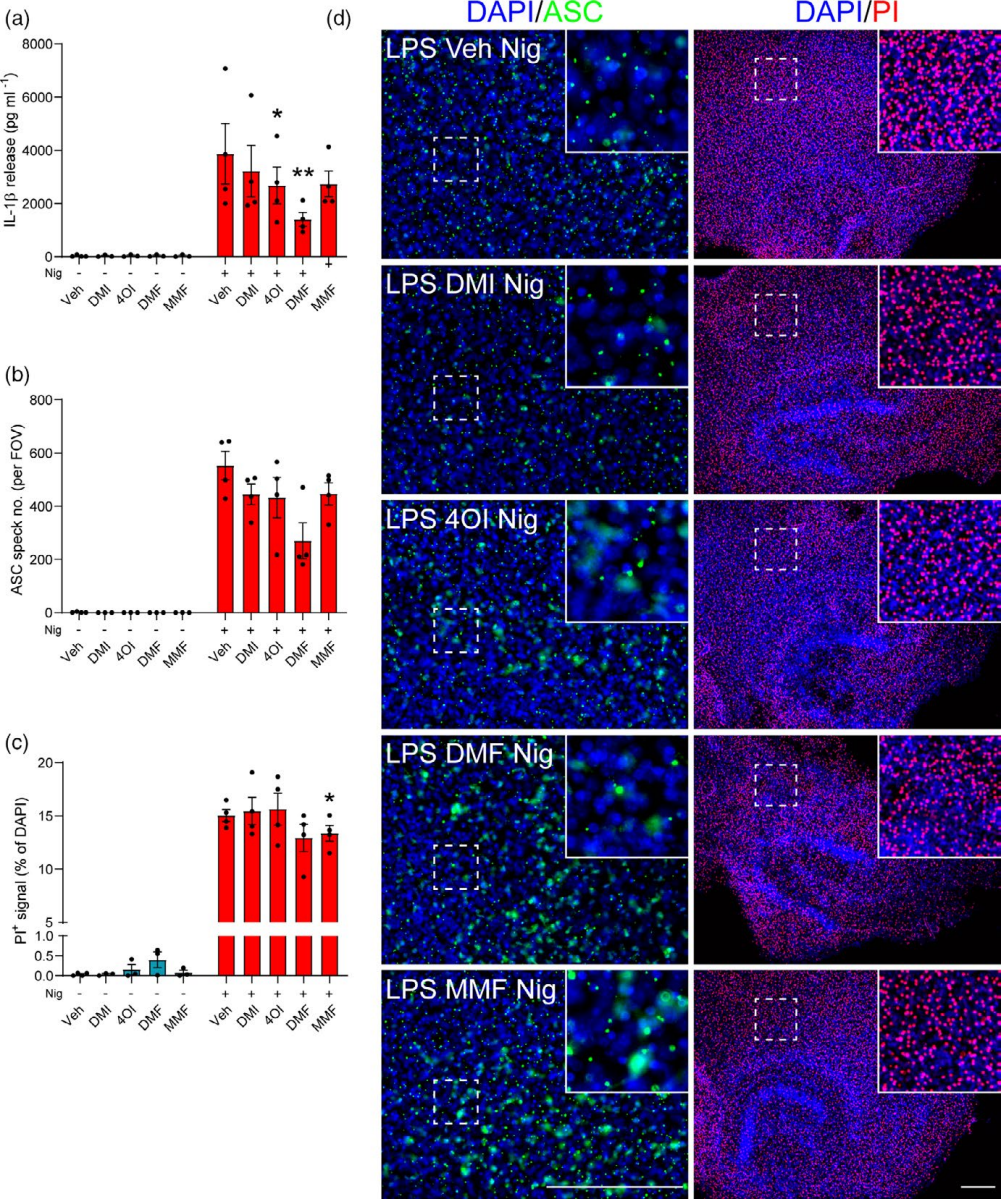
Itaconate and fumarate derivatives partly inhibit canonical NLRP3 activation in LPS‐primed OHSCs. WT OHSCs were primed with LPS (1 µg ml^−1^, 3 h) before treatment with vehicle (DMSO), DMI, 4OI, DMF (125 µM) or MMF (500 µM, 15 min). Vehicle (ethanol) or nigericin was then added to the well (10 µM, 90 min; *n* = 3–4). Propidium iodide (PI; red, 25 µg ml^−1^) was added for the final 30 min of nigericin treatment. (a) Supernatants were assessed for IL‐1β content. OHSCs were probed for nuclei (DAPI, blue) and ASC (green) by immunofluorescence staining. (b) ASC speck number per field of view was quantified. (c) The area of PI‐positive staining was determined and is expressed as a % of total area of DAPI staining. (d) Representative images are shown. Images were acquired using widefield microscopy at 20× (ASC) and 5× (PI) magnification. Scale bars are 200 µm. Data are presented as mean ± SEM. Data were analysed using mixed‐effects model (a,b,c) with Dunnett's post hoc test (vs. Veh treatment within each group). *p < 0.05; **p < 0.01. DMF, dimethyl fumarate; DMI, dimethyl itaconate; LDH, lactate dehydrogenase; LPS, lipopolysaccharide; MMF, monomethyl fumarate; OHSC, organotypic hippocampal slice culture; WT, wild‐type

Similarly, LPS‐primed OHSCs were treated with DMI, 4OI, DMF and MMF prior to nigericin stimulation. The compounds alone did not exhibit any toxicity, nor did they induce ASC speck formation (Figure S10). Following nigericin stimulation, 4OI and DMF significantly reduced IL‐1β release, but had no significant effect on ASC speck formation although there was an apparent reduction (Figure 5a, b). Only MMF treatment significantly reduced nigericin‐induced cell death, although this reduction was modest (Figure 5c). Representative immunofluorescence images of ASC speck formation are shown (Figure 5d). Together, these data suggested that the itaconate and fumarate derivatives reduced microglial NLRP3 responses.

### Itaconate and fumarate derivatives inhibit NLRP3 activation in response to LPC stimulation

**FIGURE 6:**
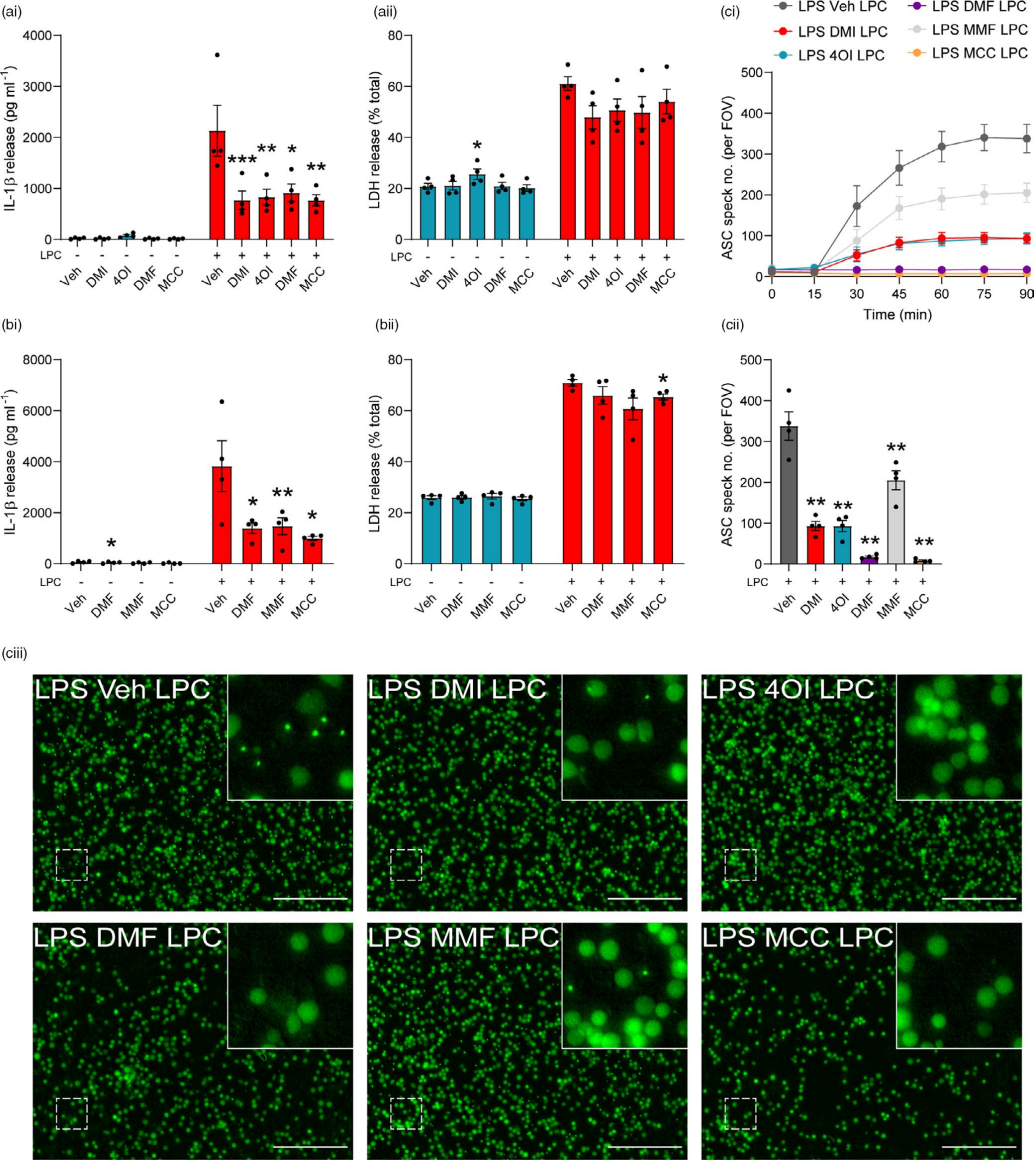
Itaconate and fumarate derivatives inhibit NLRP3 activation in response to LPC stimulation. (a) WT BMDMs were primed with LPS (1 µg ml^−1^, 4 h) before treatment with vehicle (DMSO), DMI, 4OI, DMF (125 µM) or MCC950 (MCC, 10 µM, 15 min). Vehicle (ethanol) or LPC (100 µM, 60 min) was then added to the well (*n* = 4). (b) WT BMDMs were LPS‐primed (1 µg ml^−1^, 4 h) before treatment with vehicle (DMSO), DMF (125 µM), MMF (500 µM) or MCC950 (10 µM, 15 min). Vehicle (ethanol) or LPC (100 µM, 60 min) was then added to the well (*n* = 4). Supernatants were assessed for (ai, bi) IL‐1β release by ELISA and (aii, bii) cell death (LDH release). (c) ASC–citrine BMDMs were treated as above, and ASC speck formation was measured over a period of 90 min (*n* = 4). Image acquisition began immediately after addition of LPC. (ci) ASC speck number per field of view was quantified over 90 min. (cii) ASC speck number and (ciii) fluorescence images after 90 min LPC treatment are shown. Scale bars are 200 µm. Data are presented as mean ± SEM. Data were analysed using repeated‐measures one‐way (cii) or two‐way (a, b) ANOVA with Dunnett's post hoc test (vs. Veh treatment within each group). *p < 0.05; **p < 0.01; and ***p < 0.001. BMDMs, bone marrow‐derived macrophages; DMF, dimethyl fumarate; DMI, dimethyl itaconate; LPS, lipopolysaccharide; MMF, monomethyl fumarate; WT, wild‐type

Lysophosphatidylcholine (also known as lysolecithin) is a lipid generated from the cleavage of phosphatidylcholine by phospholipase A_2_, or by the action of lecithin‐cholesterol acyltransferase [40]. LPC levels are regulated by the enzyme lysophosphatidylcholine acyltransferase, which converts LPC back to phosphatidylcholine [40]. Despite its presence during normal physiology, LPC is able to induce demyelination in experimental models of multiple sclerosis [31, 32, 33], implicating endogenous LPC dysregulation as a potential factor in multiple sclerosis pathology, although evidence of phospholipase A_2_ involvement in multiple sclerosis patients is unclear [41]. LPC can also activate the NLRP3 and NLRC4 inflammasomes in macrophages, microglia and astrocytes [42], and NLRP3 is reported to be detrimental in experimental autoimmune encephalomyelitis [36, 43, 44]. Thus, LPC‐induced NLRP3 inflammasome activation may influence LPC‐induced demyelination. We assessed whether the itaconate and fumarate derivatives could inhibit NLRP3 activation driven by LPC stimulation in macrophages. LPS‐primed BMDMs were treated with DMI, 4OI, DMF or MCC950, prior to LPC stimulation. Each of the derivatives inhibited LPC‐induced IL‐1β release to a similar extent as MCC950, indicating inhibition of NLRP3 activation, although no reductions in cell death were observed by any treatment (Figure 6ai, aii). Similarly, treatment with MMF inhibited IL‐1β release induced by LPC to the same extent as MCC950 (Figure 6bi, bii). Each of these drug treatments also reduced the formation of ASC specks in response to LPC stimulation (Figure 6ci‐ciii). To assess whether the derivatives could inhibit LPC‐induced NLRP3 activation in mixed glia, LPS‐primed mixed glia were treated with DMI, 4OI, DMF, MMF or MCC950 prior to LPC stimulation. All of the derivatives and MCC950 reduced IL‐1β release in response to LPC, and although these reductions were only statistically significant for MMF, upon normalization each of the derivatives had a significant effect, and there was no inhibition of LPC‐induced cell death (Figure S11). These data suggest that itaconate and fumarate derivatives inhibited NLRP3 activation in peripheral macrophages and microglia in response to LPC stimulation.

### Itaconate and fumarate derivatives inhibit pro‐IL‐1α cleavage

**FIGURE 7:**
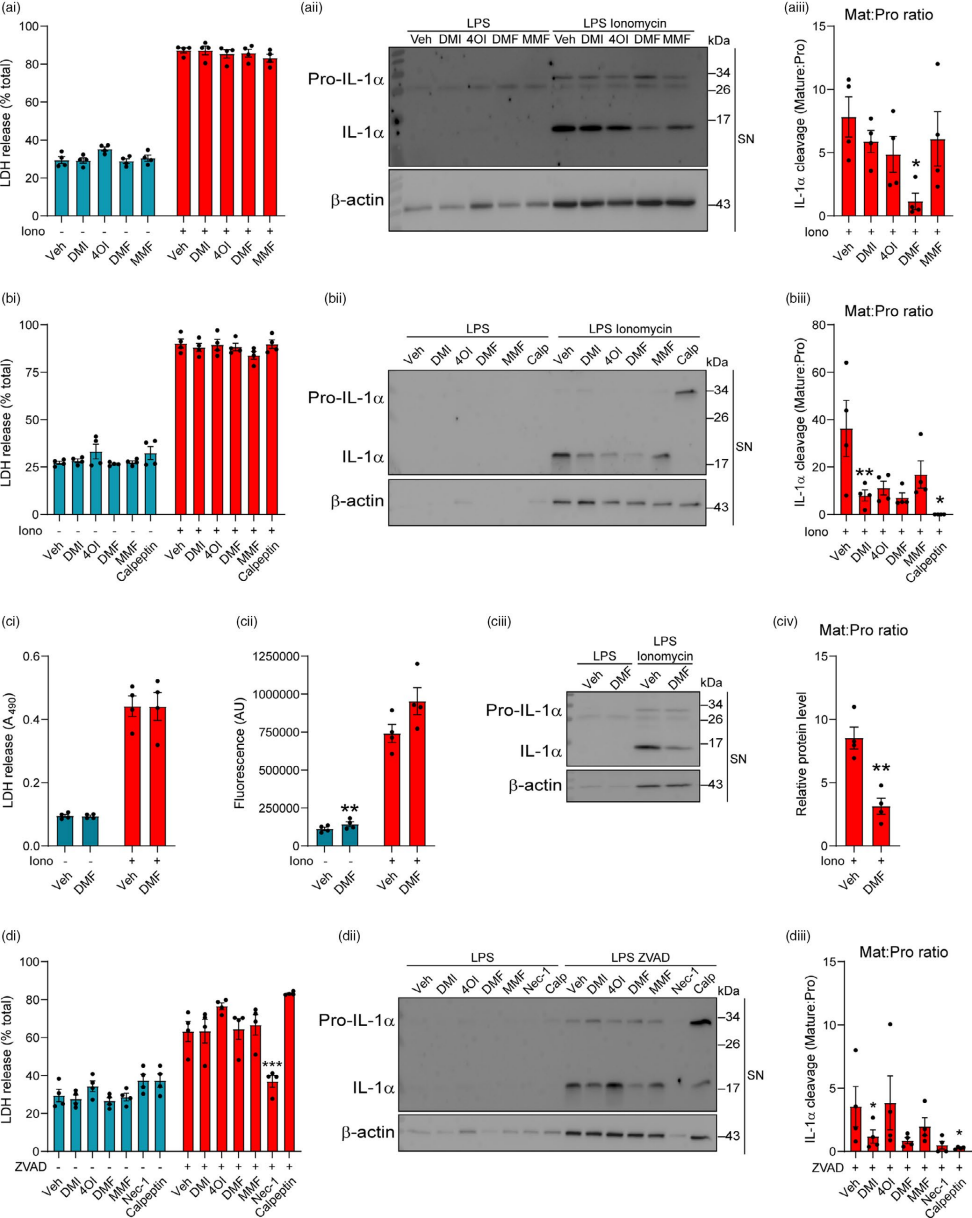
Itaconate and fumarate derivatives inhibit pro‐IL‐1α cleavage in response to ionomycin stimulation. (a–b) WT BMDMs were primed with LPS (1 µg ml^−1^, 4 h) before treatment with (a) vehicle (DMSO), DMI, 4OI, DMF (125 µM) or MMF (500 µM) for 15 min or (b) vehicle, DMI, 4OI (125 µM), DMF (30 µM), MMF (500 µM) or calpeptin (40 µM) for 3 h. Vehicle (ethanol) or ionomycin (10 µM, 1 h) was then added to the well (*n* = 4). (ai, bi) Supernatants were assessed for cell death (LDH release). (aii, bii) Supernatants were probed by Western blotting for IL‐1α protein, and (aiii, biii) the ratio of mature IL‐1α:pro‐IL‐1α is shown. For densitometry, see Figure S12bi, bii, ci, cii. (c) WT BMDMs were primed with LPS (1 µg ml^−1^, 4 h) before treatment with vehicle (DMSO) or DMF (125 µM) for 1 h. Vehicle or ionomycin (10 µM, 5 min) was then added to the well (*n* = 4). (ci) Supernatants were assessed for cell death. (cii) Cell lysates were assessed for calpain activity using a fluorescent calpain substrate. (ciii) Supernatants were probed by Western blotting for IL‐1α protein, and (civ) the ratio of mature IL‐1α:pro‐IL‐1α is shown. For densitometry, see Figure S12D. (d) WT BMDMs were primed with LPS (1 µg ml^−1^, 4 h) before treatment with vehicle, DMI, 4OI (125 µM), DMF (30 µM), MMF (500 µM), necrostatin‐1 (50 µM) or calpeptin (40 µM) for 15 min. Vehicle or ZVAD (50 µM, 5 h) was then added to the well (*n* = 4). (di) Supernatants were assessed for cell death. (dii) Supernatants were probed by Western blotting for IL‐1α protein, and (diii) the ratio of mature IL‐1α:pro‐IL‐1α is shown. For densitometry, see Figure S13. Data are presented as mean ± SEM. Data were analysed using repeated‐measures one‐way (aiii, biii, diii) or two‐way (ai, bi, ci, cii, di) ANOVA with Dunnett's (vs. Veh treatment within each group) or Sidak's post hoc test, or using unpaired *t*‐test (civ). *p < 0.05; **p < 0.01; and ***p < 0.001. BMDMs, bone marrow‐derived macrophages; DMF, dimethyl fumarate; DMI, dimethyl itaconate; LPS, lipopolysaccharide; MMF, monomethyl fumarate; WT, wild‐type

IL‐1α is a member of the IL‐1 cytokine family that is closely related to IL‐1β, and is processed into its mature form by calcium‐activated calpain proteases [45]. NLRP3 activation has been shown to induce calcium influx via gasdermin D pores, leading to calpain activation and pro‐IL‐1α cleavage [46]. Both DMI and DMF dose‐dependently inhibited IL‐1α release in response to nigericin treatment, whereas 4OI increased IL‐1α release at higher doses, likely due to increases in cell death at these doses (Figures S12A and S4). Given that 4OI has been shown to target a wide range of proteins including calpains [47], we investigated whether the itaconate and fumarate derivatives might directly affect pro‐IL‐1α processing independent of NLRP3 activation by using ionomycin, an established inducer of calpain activation and pro‐IL‐1α cleavage [45]. LPS‐primed BMDMs were treated with DMI, 4OI, DMF or MMF for either 15 min (Figure 7a) or 3 h (Figure 7b) before stimulation with ionomycin, and supernatants were assessed for the presence of cleaved IL‐1α by Western blot. None of the derivatives inhibited cell death induced by ionomycin following either derivative treatment duration (Figure 7ai, bi). Cleavage of pro‐IL‐1α into its mature form was reduced more strongly following 3‐h pretreatment, with DMF having the strongest effect at both treatment durations (Figure 7aii, aiii, bii, biii; Figure S12B, C). These reductions in pro‐IL‐1α cleavage were independent of effects on pro‐IL‐1α levels during the 3‐h drug treatment, although DMF did cause a modest but not significant reduction (Figure S12Ciii, Civ).

Given that DMF exhibited the strongest inhibition of pro‐IL‐1α cleavage, we assessed the effects of DMF on calpain activity. LPS‐primed BMDMs were treated with DMF prior to ionomycin stimulation for 5 min to induce calpain activation whilst avoiding complete cell lysis, minimizing potential loss of calpain from the cells. The cell lysates were then assessed for calpain activity, and the supernatants for the release and cleavage of pro‐IL‐1α and cell death. DMF was not able to inhibit ionomycin‐induced cell death or calpain activity, measured by cleavage of a fluorescent calpain substrate, but DMF maintained its reduction in pro‐IL‐1α cleavage (Figure 7ci‐iv; Figure S12D). This suggests that DMF may be inhibiting pro‐IL‐1α cleavage independently of a direct, irreversible effect on calpain. However, these data do not exclude an indirect effect of DMF on calpain activity that may be lost upon cell lysis.

To further explore the derivative‐mediated reduction in pro‐IL‐1α cleavage, we assessed the effects of the derivatives on necroptosis, a form of cell death that can be induced by caspase‐8 inhibition that leads to calpain‐dependent pro‐IL‐1α cleavage in LPS‐primed cells [48]. Whilst the derivatives did not inhibit ZVAD‐induced cell death, cleavage of pro‐IL‐1α was reduced by DMI and DMF (Figure 7di‐iii, Figure S13A, B). The RIPK1 inhibitor necrostatin‐1 blocked both ZVAD‐induced cell death and pro‐IL‐1α cleavage, whereas the calpain inhibitor calpeptin did not block cell death but reduced pro‐IL‐1α cleavage (Figure 7di‐iii). These reductions in pro‐IL‐1α cleavage were independent of effects on pro‐IL‐1α levels (Figure S13C, D). These data suggest that the itaconate and fumarate derivatives do not inhibit necroptosis per se, but they are able to inhibit specific events downstream of necroptosis, such as pro‐IL‐1α cleavage.

## DISCUSSION

Macrophage metabolism has emerged as an important regulator of inflammatory responses [16]. In particular, exogenous treatment with derivatives of the metabolite itaconate inhibits the expression of NF‐κB secondary response genes such as IL‐1β and IL‐6 through NRF2 accumulation [20] and inhibition of IκBζ translation [22]. However, the direct effect of itaconate derivative treatment on NLRP3 inflammasome activity is unclear, with a few recent studies indicating inhibition of IL‐1β release independently of reductions in pro‐IL‐1β [23, 28]. We confirmed that DMI, 4OI and DMF were able to inhibit the expression of pro‐inflammatory genes in macrophages upon LPS priming and show that this mechanism may have relevance in microglia. We also found that DMI, 4OI, DMF and MMF could directly inhibit biochemical hallmarks of NLRP3 inflammasome activation independently of their effects on inflammasome priming, including in human macrophages. These findings highlight that itaconate and fumarate derivatives could potentially be manipulated therapeutically in NLRP3‐driven diseases, conferring the dual benefit of targeting both priming and activation of NLRP3.

Commonly used itaconate derivatives, such as DMI and 4OI, may not fully reflect the physiology of endogenous itaconate due to differences in their properties [23]. For example, treatment with unmodified itaconate does not appear to strongly drive NRF2 signalling, nor does it inhibit IκBζ translation [23]. Nevertheless, any therapeutic potential of these derivatives maintains importance. Interestingly, Swain et al. (2020) suggested that itaconate derivatives, including unmodified itaconate, can inhibit NLRP3 activation independently of effects on NRF2 and priming. However, itaconate treatments were only applied prior to LPS priming, instead of treating LPS‐primed BMDMs with itaconate prior to NLRP3 activation. Thus, it remains possible that other effects of itaconate pretreatment, such as reduction in NLRP3 protein levels during LPS priming, could have limited IL‐1β release. We have addressed this in the current study, complementing observations that 4OI specifically inhibited NLRP3 activation in LPS‐primed cells through direct interaction with cysteine 548 on murine NLRP3, preventing NEK7 binding [28]. This mechanism is plausible, given that DMI, 4OI and DMF are electrophiles that modify cysteine residues on target proteins including KEAP1 [20, 49] and GAPDH [50, 51]. Therefore, we suspect that both the itaconate and fumarate derivatives may be working through inhibition of NLRP3 in addition to gasdermin D [19, 47].

We suggest that the inhibitory mechanisms of itaconate and fumarate derivatives on priming and activation of the canonical NLRP3 inflammasome may be consistent in microglia. Thus, the implications for immunometabolic regulation of inflammasome responses in the brain should be explored further, particularly in the context of brain pathology. Transcriptomic databases indicate that microglia exhibit relatively high expression of *Nfe2l2* (NRF2) and *Keap1* [52, 53]. The itaconate‐synthesizing enzyme *Irg1* (also known as *Acod1*) was recently shown to be upregulated in response to LPS treatment in OHSCs [54], driving subsequent itaconate production, and this was prevented by microglial depletion, indicating that this is primarily a microglial response. Furthermore, treatment with 4OI limited LPS‐induced IL‐6 production, suggesting that itaconate derivatives can exhibit anti‐inflammatory effects in OHSCs [54].

Whilst mixed glia and OHSCs are useful tools to study microglial function, further studies are required to fully understand the physiological relevance of microglial metabolic reprogramming in vivo. NRF2 activation is associated with a protective effect in experimental ischaemic stroke models [55], and IRG‐deficient mice exhibit exacerbated brain damage to acute ischaemic stroke [56]. These studies suggest that itaconate production could be an endogenous, protective response to limit ischaemic damage. Our previous report showed increased levels of IL‐1β and NLRP3 expression after ischaemic stroke, but NLRP3 deficiency or inhibition did not improve stroke outcome [57]. This could suggest that endogenous itaconate production within the brain in response to ischaemia, whilst too late to inhibit inflammatory cytokine production, may be able to limit NLRP3 inflammasome activation. This is supported by a recent study in which endogenous itaconate was shown to accumulate in macrophages upon LPS priming and confer tolerance to downstream NLRP3 activation [58]. It is important to note that detection of increased NRF2 levels in response to DMI and 4OI treatment was not as reliable in the microglial models employed in this study compared with DMF treatment, likely indicating a lower amount of NRF2 accumulation. The reduction in pro‐IL‐1β levels was also weaker in the mixed glia and OHSCs compared with the BMDMs, perhaps due to the presence of other cell types such as astrocytes, oligodendrocytes and neurons, which differentially express NRF2 [52, 53]. It should also be noted that the extent of NLRP3 inhibition mediated by the itaconate and fumarate derivatives in the OHSCs was lower than in the BMDM and mixed glial assays. It is possible that higher doses or longer treatment times of the metabolite derivatives would result in greater NLRP3 inhibition, given that we have previously shown that MCC950 potently inhibits IL‐1β release and ASC speck formation in OHSCs, indicating that pharmacological inhibition of NLRP3 in OHSCs can be achieved with efficacious compounds [30].

We demonstrate that DMF, an approved clinical treatment for relapsing–remitting multiple sclerosis [49, 59, 60] and psoriasis [61], was an effective NLRP3 inhibitor in both macrophages and microglia, as has been suggested previously [26, 27, 62]. We also showed that DMF, as well as DMI and 4OI, inhibited NLRP3 activation in response to LPC stimulation of macrophages, a demyelinating agent that can drive NLRP3 and NLRC4 activation in macrophages, microglia and astrocytes [42]. The mechanism underlying DMF's beneficial effect in multiple sclerosis and psoriasis is unclear and is commonly suggested to be mediated via NRF2 activation, although NRF2‐independent effects are also reported [63]. Given that DMF is able to potently inhibit NLRP3 activation and directly inhibit gasdermin D cleavage [19] and that NLRP3 is detrimental in the experimental autoimmune encephalomyelitis model of multiple sclerosis [36, 43, 44], it is possible that DMF's protective response in multiple sclerosis is in part mediated through dampened microglial and macrophage NLRP3 activation that may promote neuronal demyelination. Inhibition of NLRP3 activation in macrophages or microglia could also facilitate other inflammatory responses that promote clearance of damaged myelin and remyelination [64]. Evidence of caspase‐1 activation and gasdermin D‐mediated pyroptosis has also been observed in the CNS of multiple sclerosis patients and in animal models [65, 66], further implicating NLRP3 involvement. Upon administration, DMF is hydrolysed to its active metabolite MMF, which can be detected in the plasma [67, 68]. Oral MMF administration was recently shown to exhibit comparable MMF plasma levels with that of oral DMF administration [69]. MMF also induces KEAP1 cysteine alkylation and NRF2 activation [49], suggesting it may exert similar inhibitory effects to DMF on the priming response. Importantly, here we confirm that MMF inhibited NLRP3 activation in response to both nigericin and LPC stimulation in macrophages, suggesting that NLRP3 inhibition could indeed be a relevant in vivo mechanism for DMF treatment. Given that DMI and 4OI, which both also drive NRF2 accumulation, exerted similar inhibitory effects on nigericin‐ and LPC‐induced NLRP3 activation, it is possible that these itaconate derivatives may also offer therapeutic potential in the development of new inhibitors for the treatment of multiple sclerosis. Indeed, DMI was recently shown to be protective in a mouse model of multiple sclerosis [70].

The itaconate and fumarate derivatives also reduced the cleavage of pro‐IL‐1α, with DMF having the strongest effect. Increased IL‐1α expression has previously been identified in a subset of disease‐associated microglia in an experimental autoimmune encephalomyelitis model [71], though whether it is important in the pathology is unknown. Mechanistically, calpain catalytic subunits have been identified as target proteins that can be modified by prolonged 4OI treatment in RAW macrophages [47]. However, we did not observe calpain inhibition by DMF, and thus, the derivatives may act directly on IL‐1α itself or may interfere with the recruitment of pro‐IL‐1α to activated calpain.

We have revealed a multifaceted mechanism of inflammatory regulation by metabolite derivatives that inhibit both the priming and activation of the canonical NLRP3 pathway, as well as discovering effects on IL‐1α processing. This suggests that treatments based on derivatives of metabolites such as itaconate and fumarate may represent a viable therapeutic strategy in NLRP3‐ and IL‐1α‐driven diseases, although further work is required to confirm this.

## CONFLICT OF INTEREST

The authors declare that the research was conducted in the absence of any commercial or financial relationships that could be construed as a potential conflict of interest.

## AUTHOR CONTRIBUTIONS

CH, EL, SMA and DB conceptualized the study. CH, EL and DB contributed to methodology. CH, EL and JPG investigated the study. CH wrote the original draft. CH, EL, JPG, SMA and DB wrote, reviewed and edited the manuscript. CH contributed to visualization. EL, SMA and DB supervised the study. DB and SMA contributed to funding acquisition.

## Supporting information

Supplementary Material
