# Supplementary material for: Itaconate and fumarate derivatives inhibit priming and activation of the canonical NLRP3 inflammasome in macrophages
Source: Immunology. 2022 Mar 2;165(4):460–80. doi: 10.1111/imm.13454 (PMC9426622; doi:10.1111/imm.13454)
Supplement: Supplementary file 1 — Supplementary Material [file IMM-165-460-s001.docx]

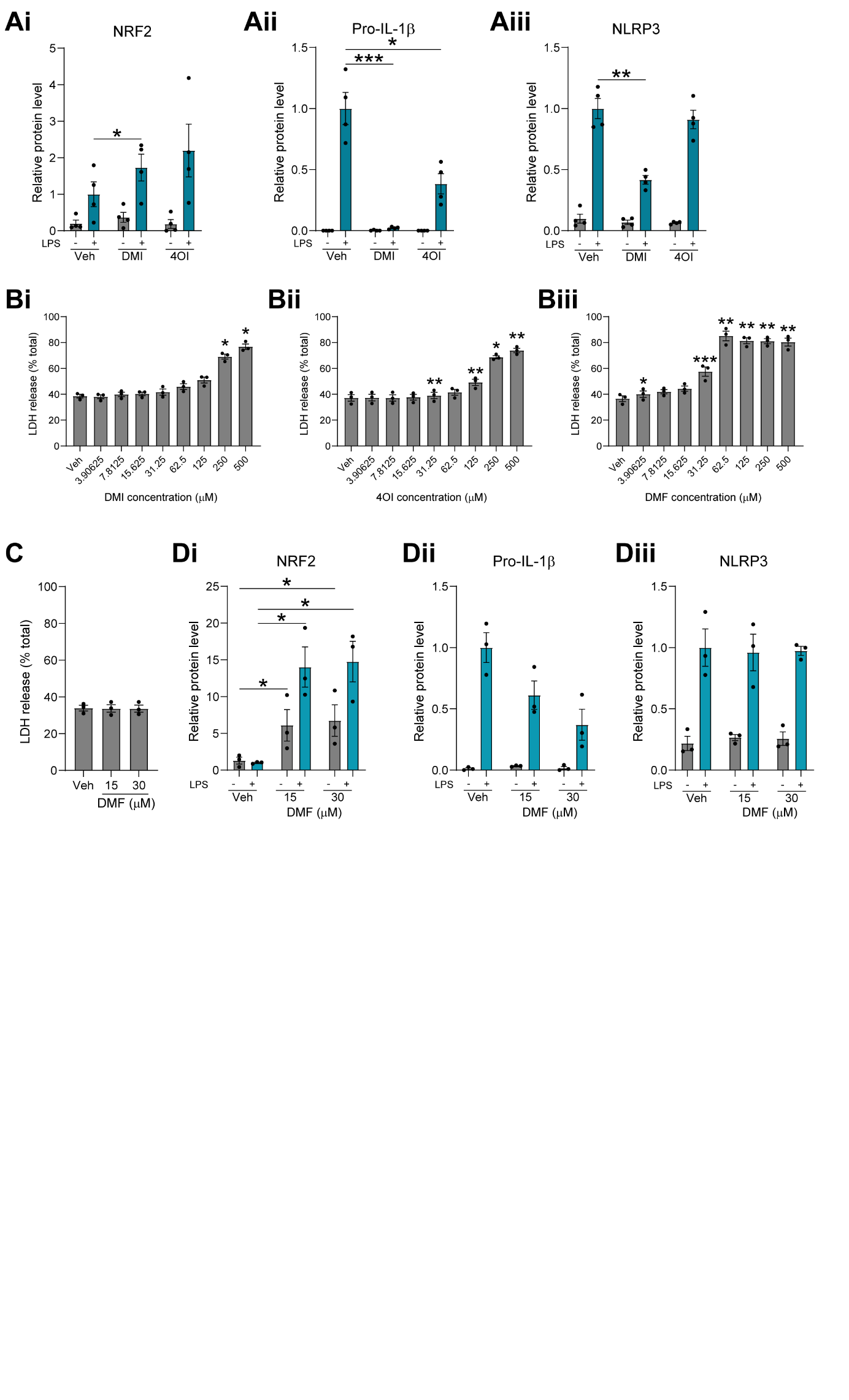


**Supplementary Figure 1.** **Densitometry analysis of western blotting from Figure 1, and toxicity of itaconate and fumarate derivatives.** (**A**) Densitometry of (**Ai**) NRF2, (**Aii**) pro-IL-1β and (**Aiii**) NLRP3 protein levels from Figure 1A (expressed relative to Veh+LPS treatment). (**B**) WT BMDMs were treated with vehicle (DMSO), (**Bi**) DMI, (**Bii**) 4OI or (**Biii**) DMF (3.90625–500 µM, 24 h). Supernatants were assessed for cell death (LDH release). (**C**) WT BMDMs were treated with vehicle (DMSO) or DMF (15 or 30 µM, 5 h). Supernatants were assessed for cell death (LDH release). (**D**) Densitometry of (**Di**) NRF2, (**Dii**) pro-IL-1β and (**Diii**) NLRP3 protein levels from Figure 1C (expressed relative to Veh+LPS treatment). Data are presented as mean ± SEM. Data were analysed using repeated-measures one-way (B, C) or two-way (A, D) ANOVA with Dunnett’s post-hoc test (versus Veh treatment within each group). *P<0.05; **P<0.01; ***P<0.001.


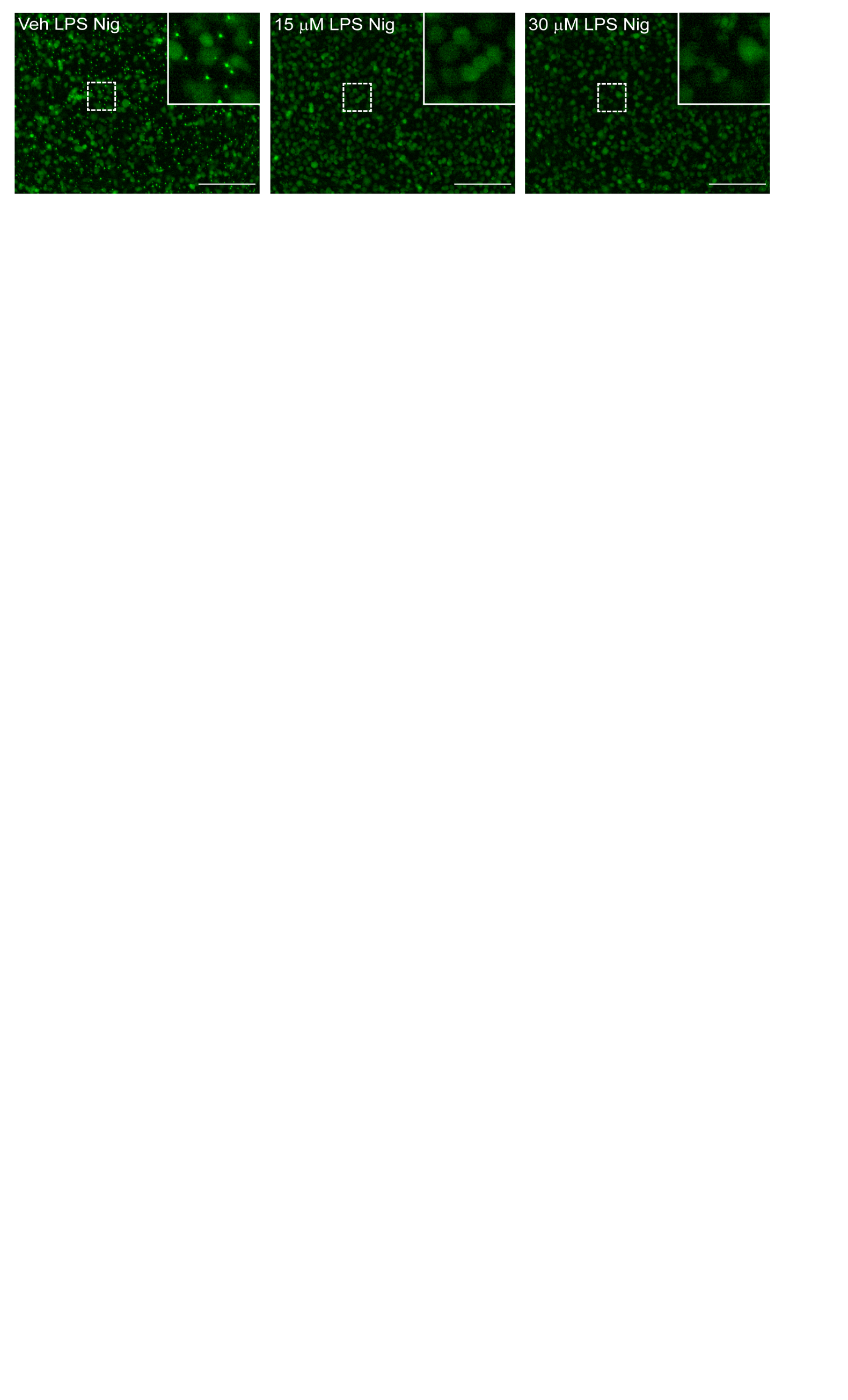


**Supplementary Figure 2. DMF pre-treatment inhibits activation of the canonical NLRP3 inflammasome.** ASC–citrine BMDMs were treated with vehicle (DMSO) or DMF (15 or 30 µM, 1 h), followed by LPS (1 µg ml^–1^, 4 h) and nigericin (10 µM, 90 min; n=4). Fluorescence images after 90 min nigericin treatment are shown. Scale bars are 200 µm.

**
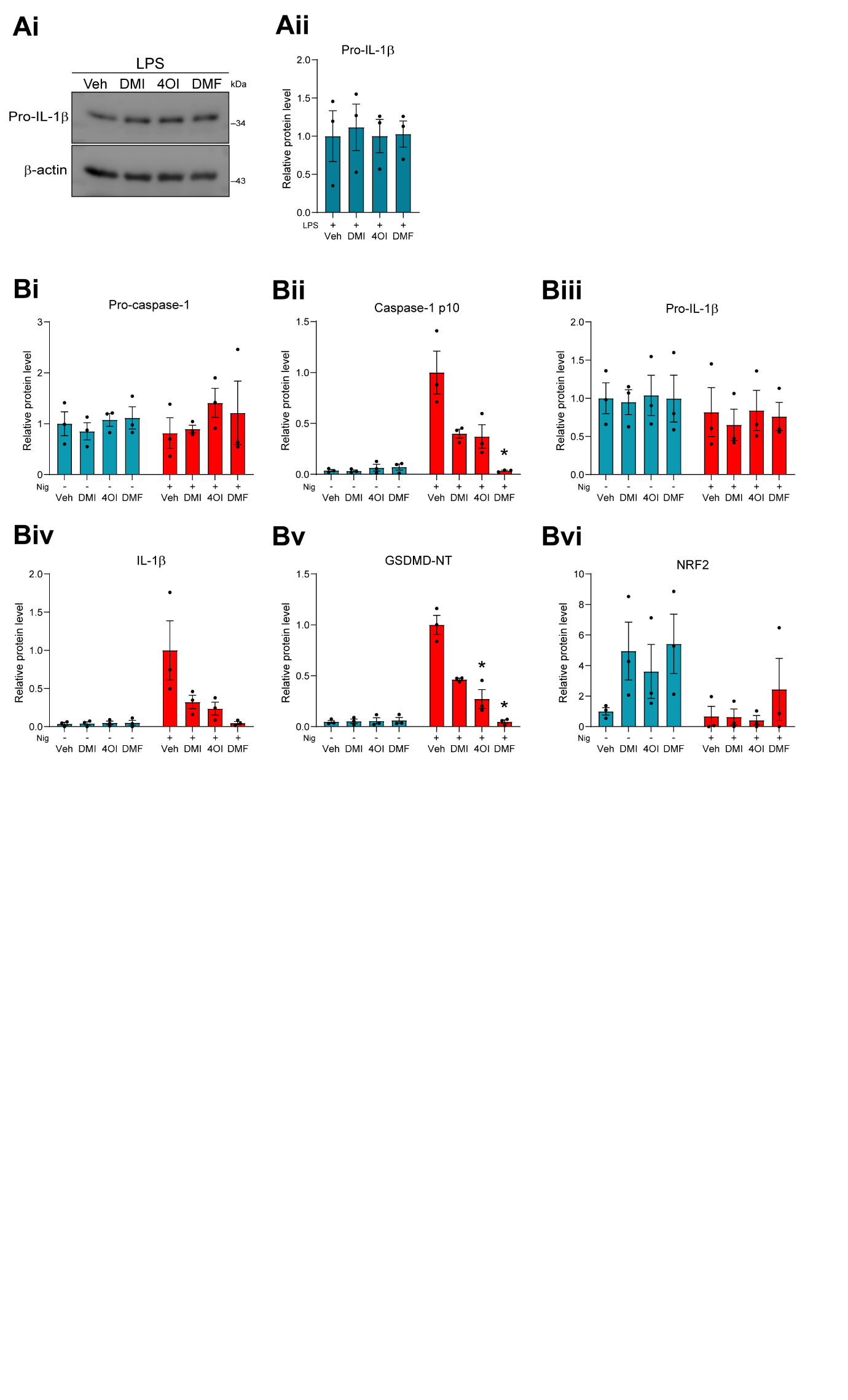
**

**Supplementary Figure 3. Densitometry analysis of western blotting from Figure 2.** (**Ai**) Cell lysates from Figure 2A were probed by western blotting for pro-IL-1β protein, and (**Aii**) densitometry of pro-IL-1β protein levels are shown (expressed relative to LPS Veh treatment). (**B**) Densitometry of several markers of inflammasome activation from Figure 2C (expressed relative to either LPS Veh or LPS Veh Nig treatment). Data are presented as mean ± SEM. Data were analysed using repeated-measures two-way ANOVA with Dunnett’s post-hoc test (versus Veh treatment within each group). *P<0.05.


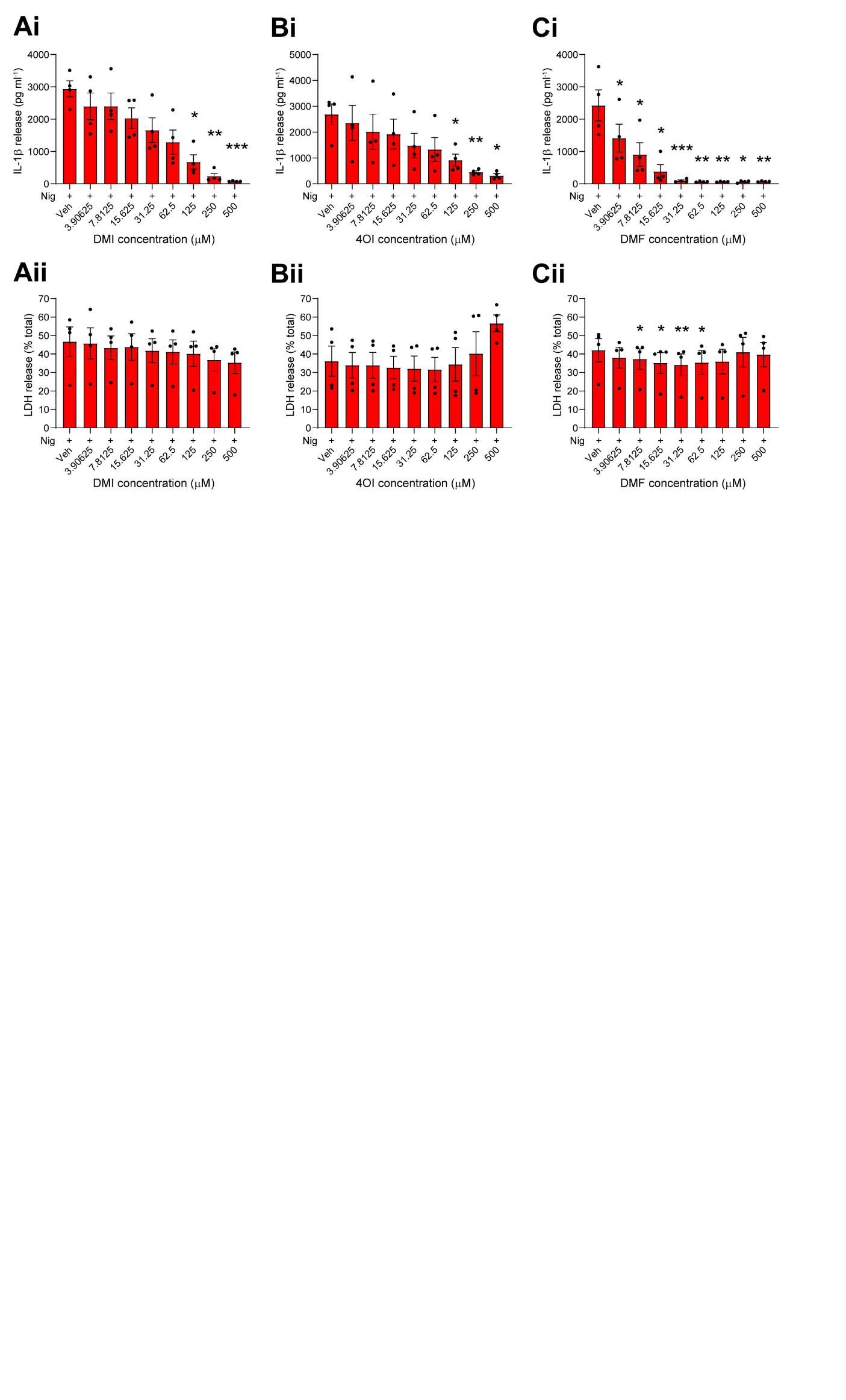


**Supplementary Figure 4.** **Itaconate and fumarate derivatives dose-dependently inhibit canonical NLRP3 inflammasome activation.** WT BMDMs were primed with LPS (1 µg ml^–1^, 4 h) before treatment with vehicle (DMSO), (**A**) DMI, (**B**) 4OI or (**C**) DMF (3.90625–500 µM, 15 min). Nigericin was then added to the well (10 µM, 60 min; n=4). Supernatants were assessed for IL‑1β content by ELISA and cell death (LDH release). Data are presented as mean ± SEM. Data were analysed using repeated-measures one-way ANOVA with Dunnett’s post-hoc test (versus Veh treatment. *P<0.05; **P<0.01; ***P<0.001.


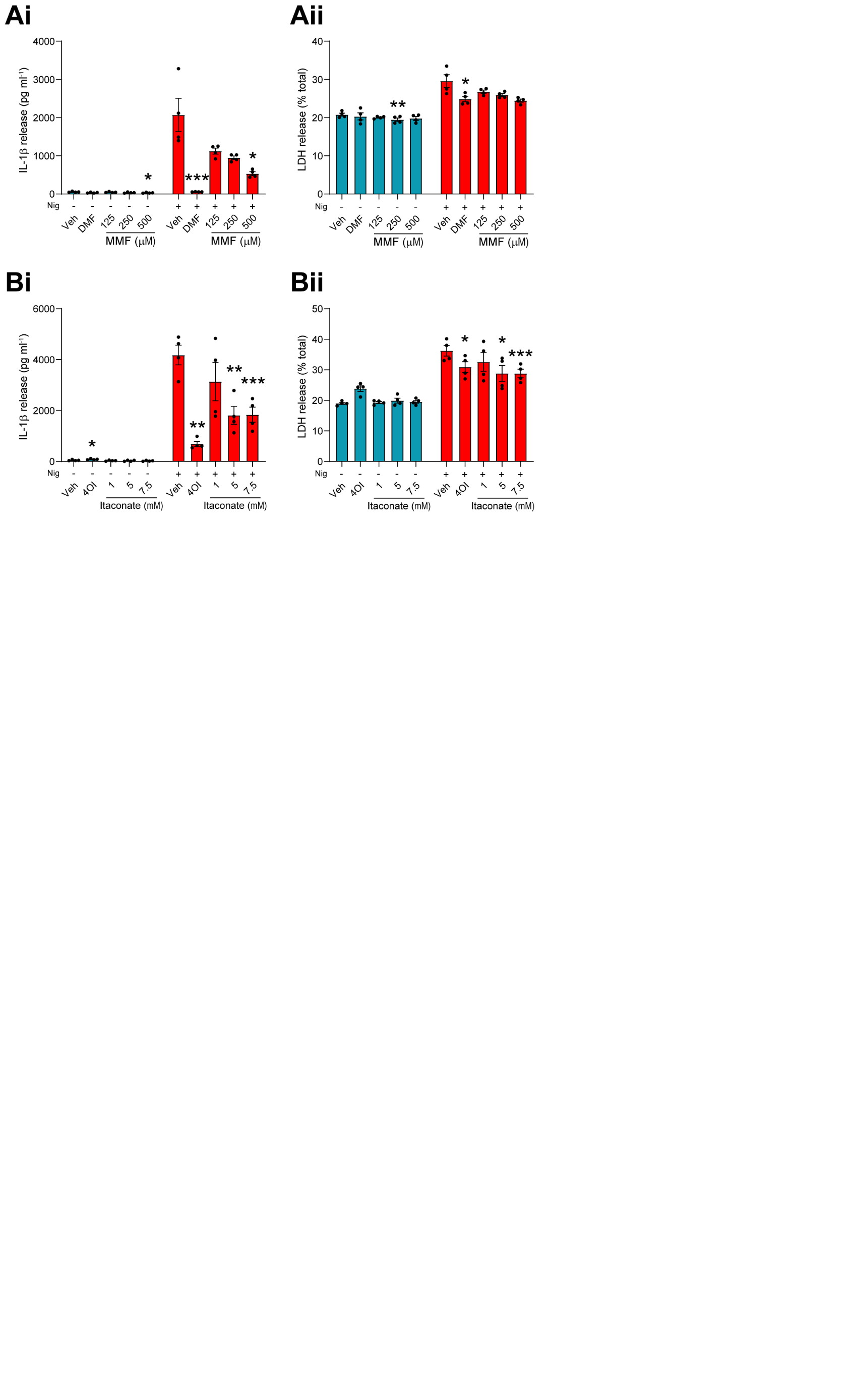


**Supplementary Figure 5.** **MMF and** **unmodified itaconate can inhibit canonical NLRP3 inflammasome activation.** (**A**) WT BMDMs were LPS primed (1 µg ml^–1^, 4 h) before treatment with vehicle (DMSO), DMF (125 µM) or MMF (125–500 µM, 15 min). Nigericin was then added to the well (10 µM, 60 min; n=4). Supernatants were assessed for (**Ai**) IL-1β release and (**Aii**) LDH release (**B**) WT BMDMs were primed with LPS (1 µg ml^–1^, 4 h) before treatment with vehicle (DMSO), 4OI (125 µM) or unmodified itaconate (1–7.5 mM, 15 min). Nigericin was then added to the well (10 µM, 60 min; n=4). Supernatants were assessed for (**Bi**) IL-1β release and (**Bii**) LDH release. Data are presented as mean ± SEM. Data were analysed using repeated-measures two-way ANOVA with Dunnett’s post-hoc test (versus Veh treatment within each group). *P<0.05; **P<0.01; ***P<0.001.


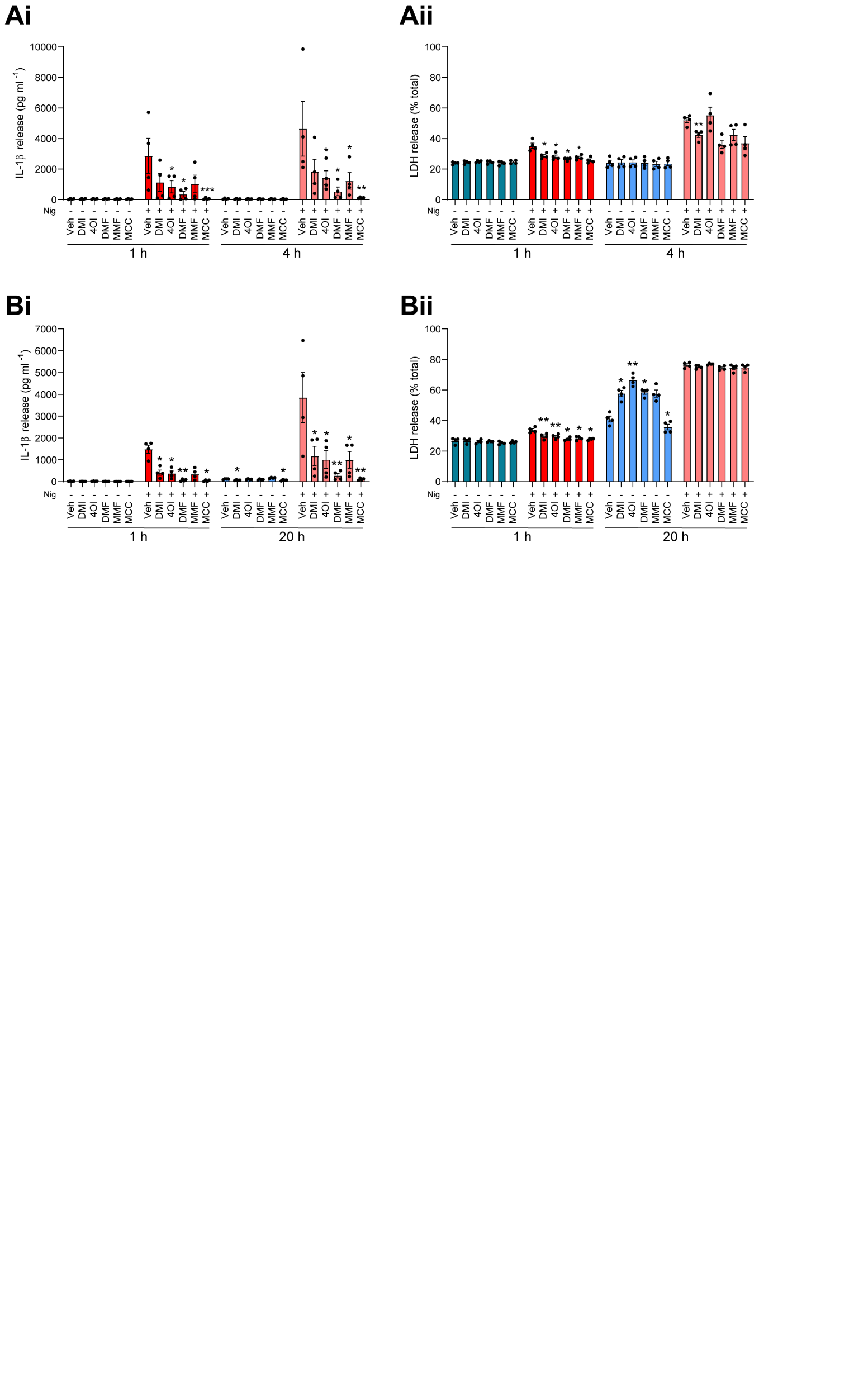


**Supplementary Figure 6.** **Itaconate and fumarate derivatives inhibit NLRP3 inflammasome activation, but not cell death, in response to prolonged nigericin stimulation.** WT BMDMs were LPS primed (1 µg ml^–1^, 4 h) before treatment with vehicle (DMSO), DMI (125 µM), 4OI (125 µM), DMF (30 µM) or MMF (500 µM, 15 min). Nigericin was then added to the well for 1 h or (**A**) 4 or (**B**) 20 h (10 µM; n=4). Supernatants were assessed for (**Ai, Bi**) IL-1β release and (**Aii, Bii**) LDH release. Data are presented as mean ± SEM. Data were analysed using repeated-measures two-way ANOVA with Dunnett’s post-hoc test (versus Veh treatment within each group). *P<0.05; **P<0.01; ***P<0.001.


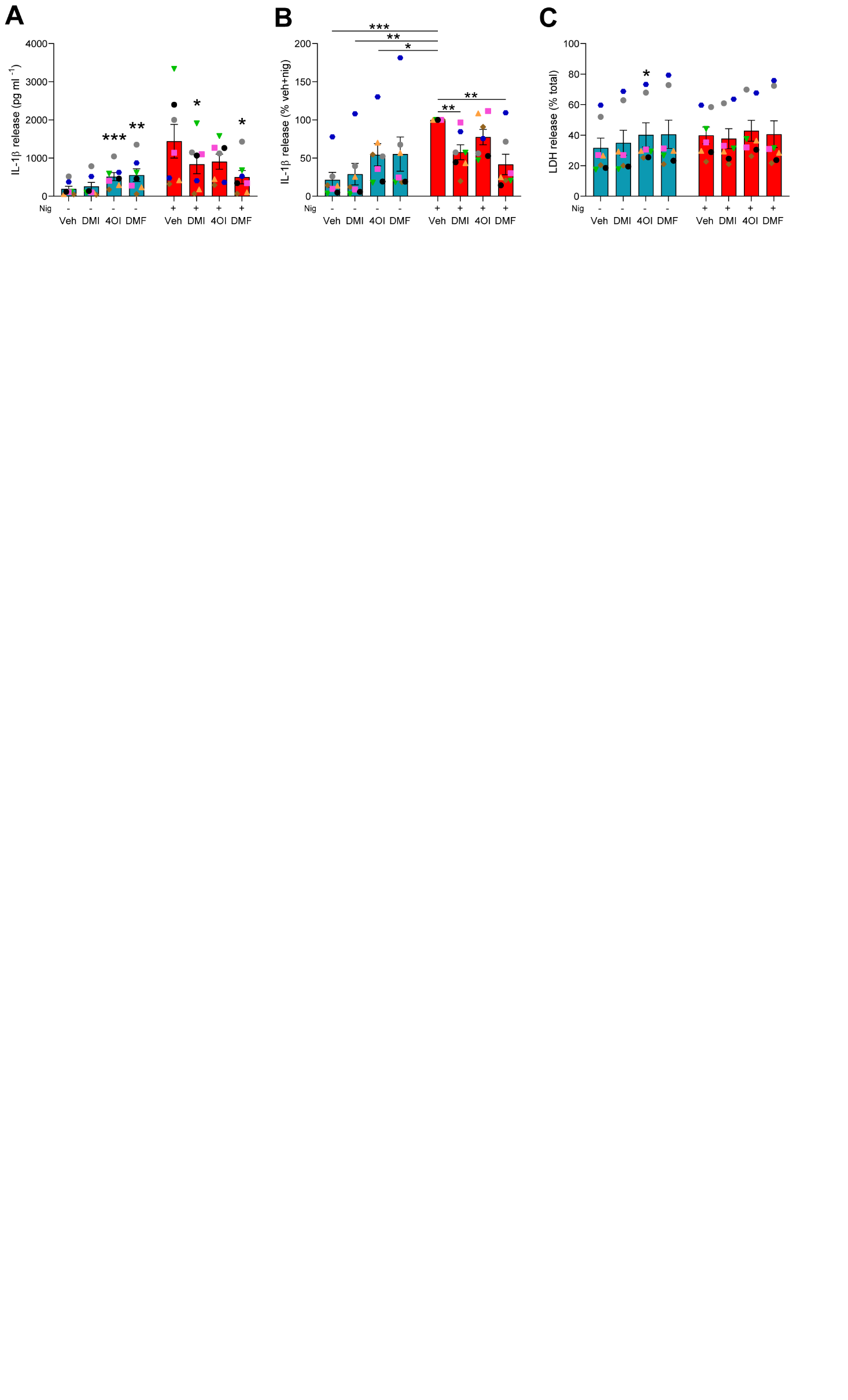


**Supplementary Figure 7.** **Itaconate and fumarate derivatives inhibit nigericin-induced NLRP3 activation in primary human MDMs.** Human MDMs were LPS primed (1 µg ml^–1^, 4 h) before treatment with vehicle (DMSO), DMI, 4OI or DMF (125 µM, 15 min). Nigericin was then added to the well (10 µM, 60 min; n=7). Supernatants were assessed for (**A**) IL-1β release, which is (**B**) also expressed as a percentage of vehicle + nig treatment, and (**C**) LDH release. Data points from each biological repeat are represented by colour and shape. Supernatants were assessed for cytokine content by ELISA. Data are presented as mean ± SEM. Data were analysed using repeated measures two-way ANOVA with Dunnett’s post-hoc test (versus Veh treatment within each group) (A, C) or one-sample t test versus a value of 100% followed by Holm-Sidak correction (B). *P<0.05; **P<0.01; ***P<0.001.

**
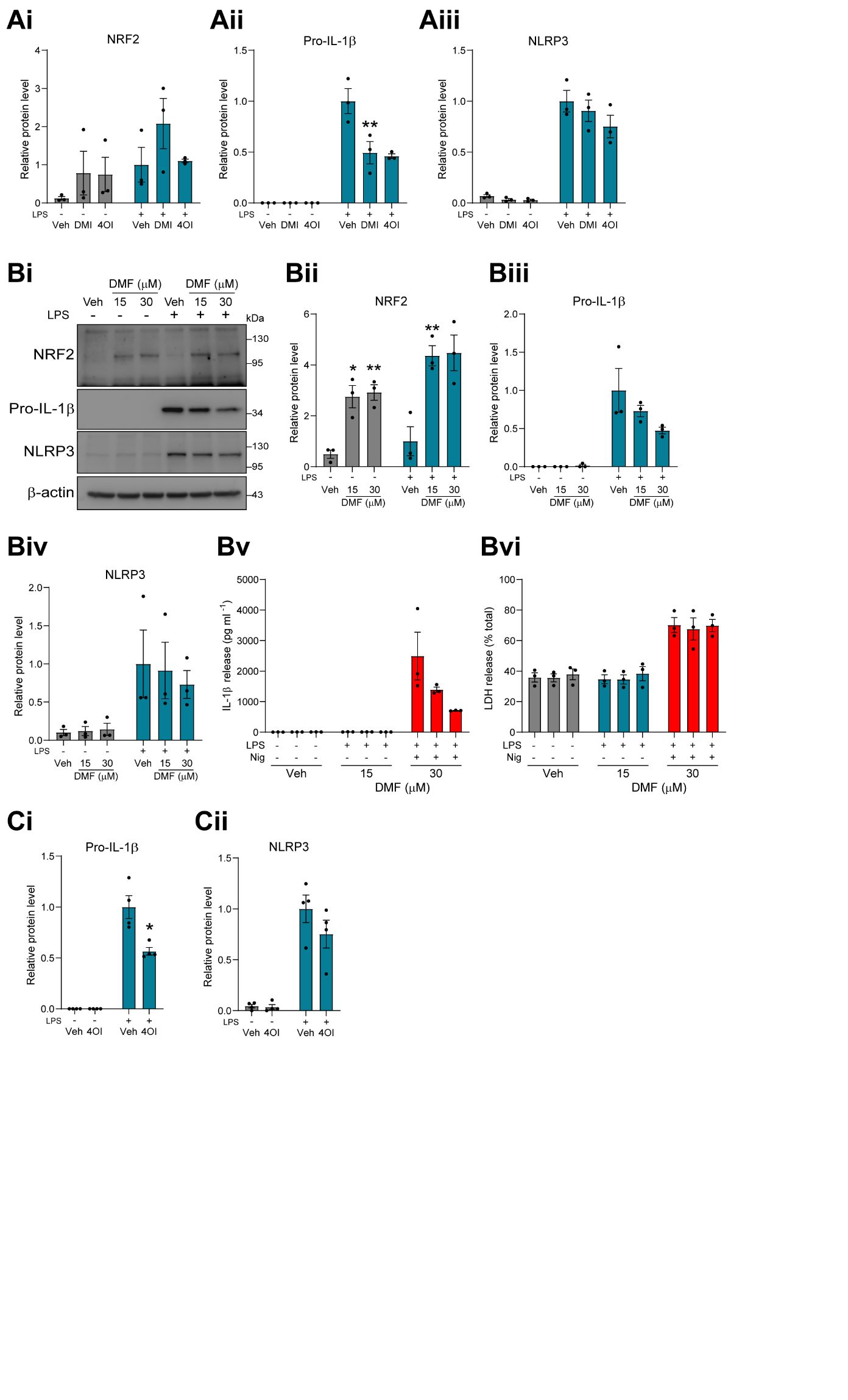
Supplementary Figure 8. Densitometry analysis of western blotting from Figure 3.** (**A**) Densitometry of NRF2, pro-IL-1β and NLRP3 protein levels from Figure 3Ai (expressed relative to LPS Veh treatment). (**B**) WT mixed glia were treated with vehicle or DMF (15 or 30 µM, 1 h). LPS (1 µg ml^–1^, 3 h) was then added to the wells to induce priming (n=3). (**Bi**) Cell lysates were probed by western blotting for NRF2, pro-IL-1β and NLRP3 protein. (**Bii-iv**) Densitometry of NRF2, pro-IL-1β and NLRP3 protein levels (expressed relative to LPS Veh treatment). (**Bv, Bvi**) WT mixed glia were treated as above, followed by nigericin (10 µM, 60 min; n=3). Supernatants were assessed for (**Bv**) IL-1β release and (**Bvi**) cell death (LDH release). (**C**) Densitometry of pro-IL-1β and NLRP3 protein levels from Figure 3Bi (expressed relative to LPS Veh treatment). Data are presented as mean ± SEM. Data were analysed using repeated-measures two-way ANOVA with Dunnett’s (A,B) or Sidak’s (C) post-hoc test (versus Veh treatment within each group). *P<0.05; **P<0.01.

**
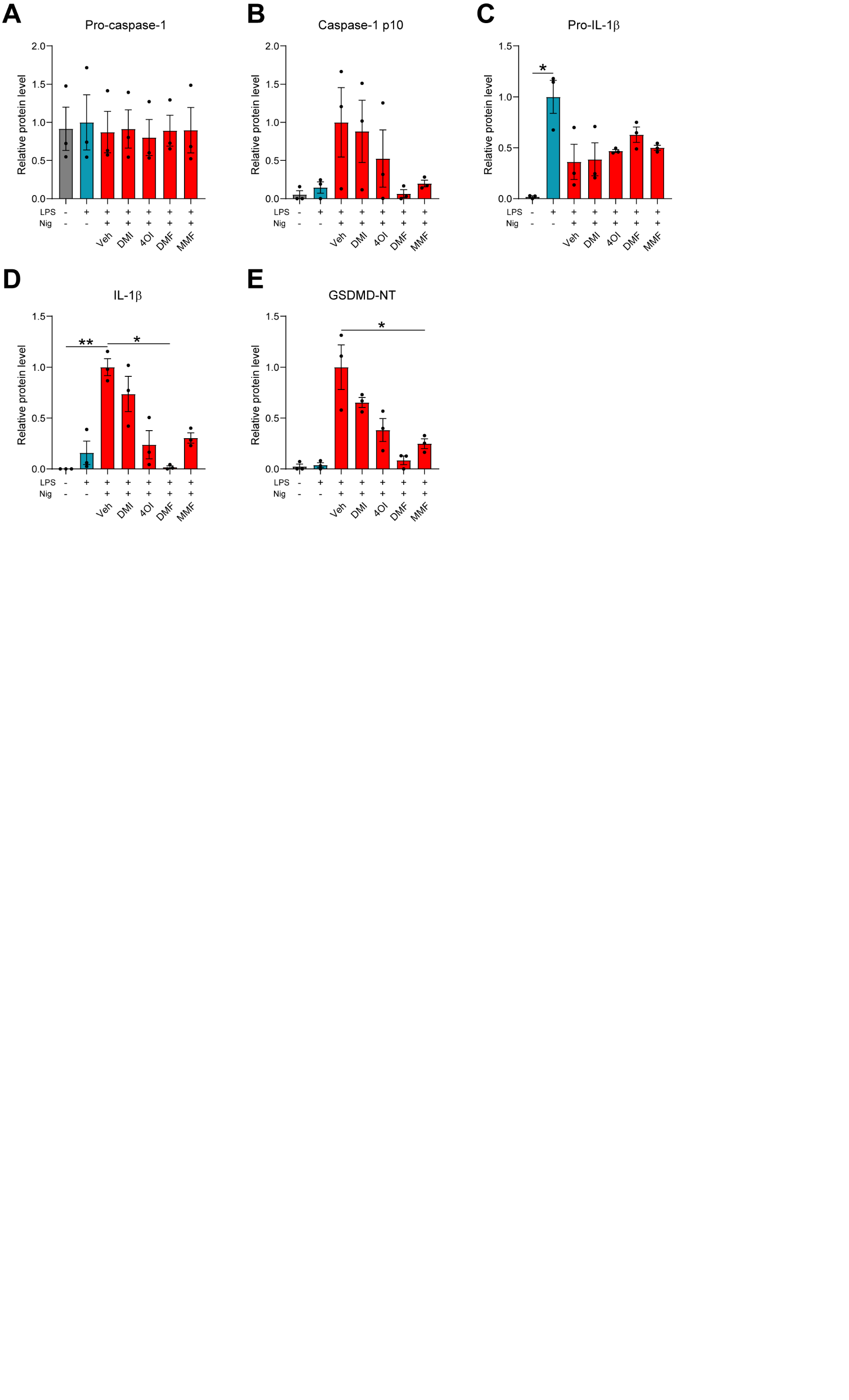
**

**Supplementary Figure 9. Densitometry analysis of western blotting from Figure 4B.** Densitometry of several markers of inflammasome activation from Figure 4B (expressed relative to either LPS + Veh or LPS + Veh + Nig treatment). Data are presented as mean ± SEM. Data were analysed using repeated-measures one-way ANOVA with Dunnett’s post-hoc test (versus LPS + Veh or LPS + Veh + Nig treatment within each group). *P<0.05; **P<0.01.


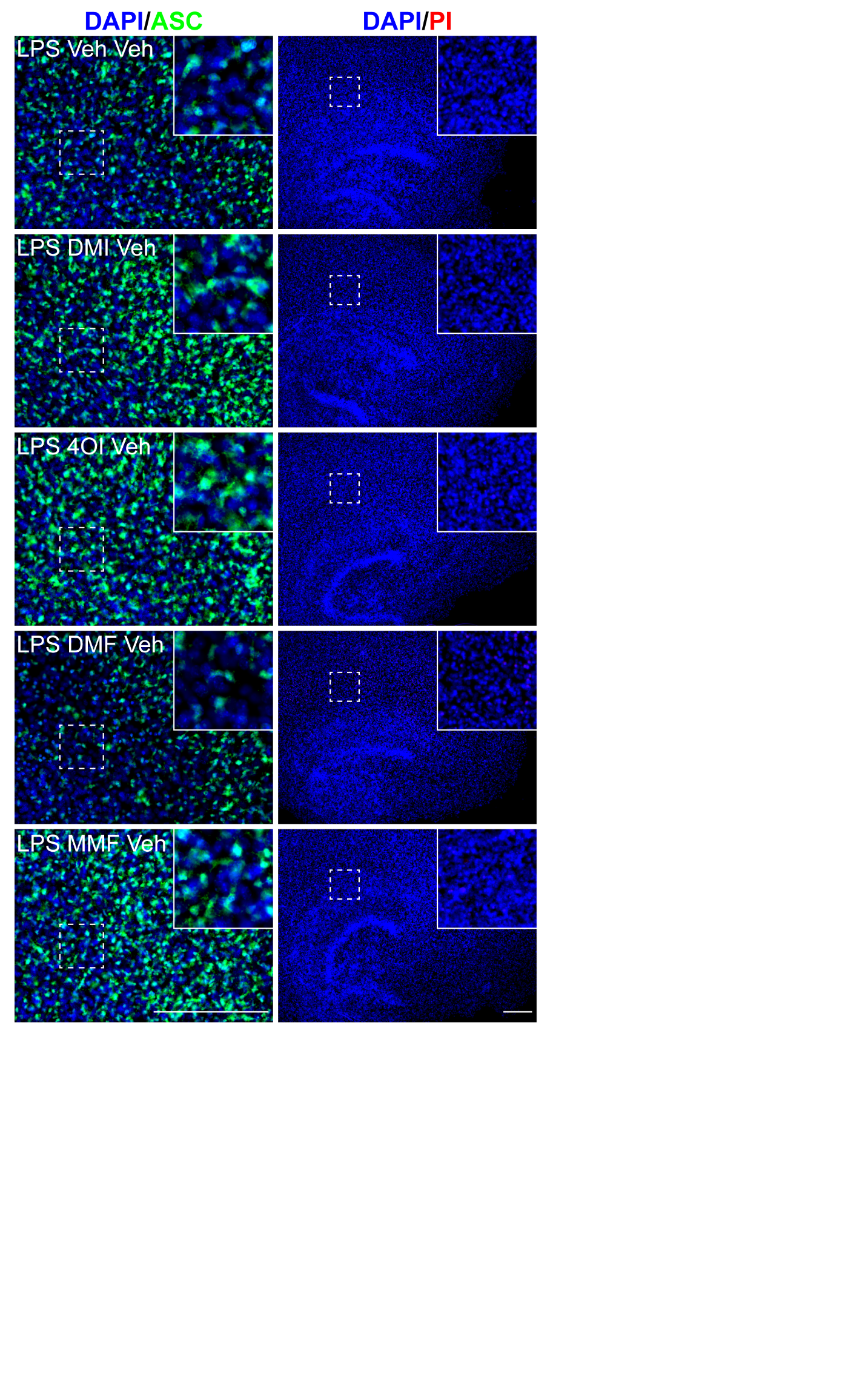


**Supplementary Figure 10.** **Representative images of vehicle-treated WT OHSCs in Figure 5.** WT OHSCs were primed with LPS (1 µg ml^–1^, 3 h) before treatment with vehicle (DMSO), DMI, 4OI, DMF (125 µM) or MMF (500 µM, 15 min). Vehicle (ethanol) was then added to the well (1:200, 90 min; n=3–4). Propodium iodide (PI; red, 25 µg ml^–1^) was added for the final 30 min of vehicle treatment. OHSCs were probed for nuclei (DAPI, blue) and ASC (green) by immunofluorescence staining. Images were acquired using widefield microscopy at 20X (ASC) and 5X (PI) magnification. Scale bars are 200 µm.


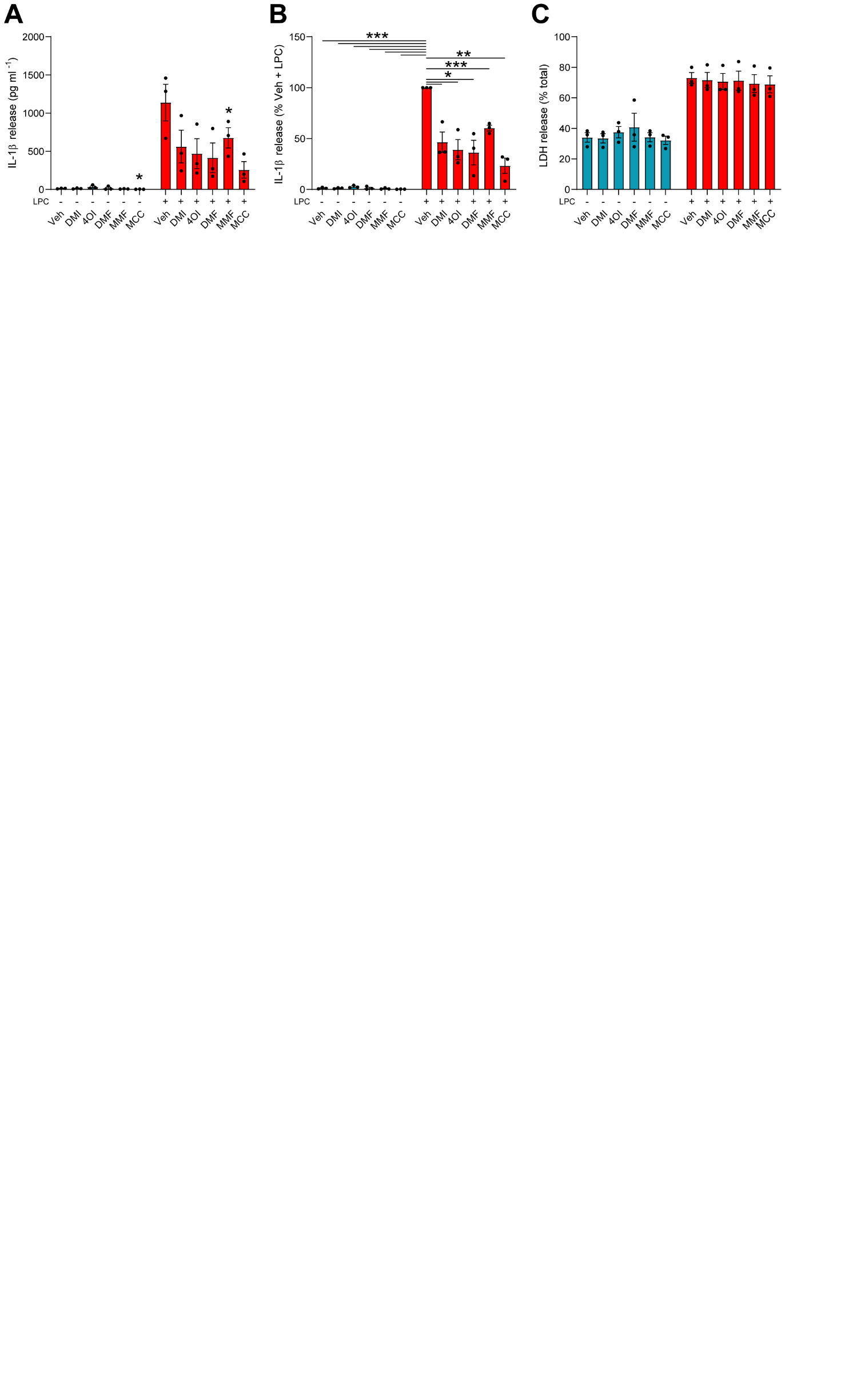


**Supplementary Figure 11.** **Itaconate and fumarate derivatives inhibit LPC-induced NLRP3 activation in mixed glia.** Mixed glia were primed with LPS (1 µg ml^–1^, 3 h) before treatment with vehicle, DMI, 4OI, DMF (125 µM), MMF (500 µM) or MCC950 (10 µM, 15 min). Vehicle or LPC (100 µM, 4 h) was then added to the well (n=3). Supernatants were assessed for (**A**) IL-1β release, which is (**B**) also expressed as a percentage of vehicle + LPC treatment, and (**C**) LDH release. Supernatants were assessed for cytokine content by ELISA. Data are presented as mean ± SEM. Data were analysed using repeated measures two-way ANOVA with Dunnett’s post-hoc test (versus Veh treatment within each group) (A, C) or one-sample t test versus a value of 100% followed by Holm-Sidak correction (B). *P<0.05; **P<0.01; ***P<0.001.


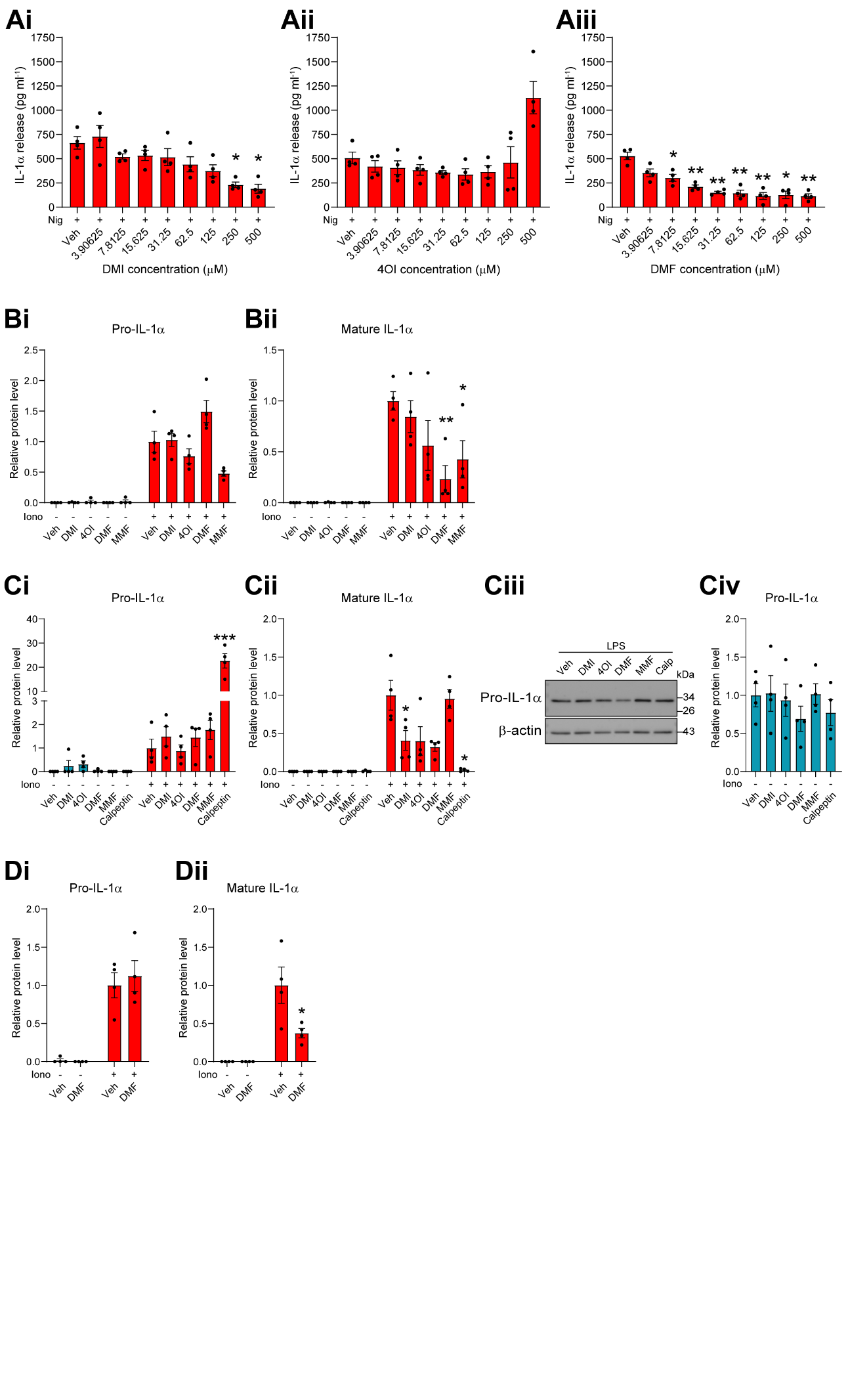


**Supplementary Figure 12. Itaconate and fumarate derivatives inhibit pro-IL-1α cleavage in response to nigericin and ionomycin stimulation.** (**A**) WT BMDMs were primed with LPS (1 µg ml^–1^, 4 h) before treatment with vehicle (DMSO), (**Ai**) DMI, (**Aii**) 4OI or (**Aiii**) DMF (3.90625–500 µM, 15 min). Nigericin was then added to the well (10 µM, 60 min; n=4). Supernatants were assessed for IL‑1α content by ELISA. For cell death, see Supplementary Figure 4. (**B**) Densitometry of (**Bi**) pro-IL-1α and (**Bii**) mature IL-1α protein levels from Figure 7Aii (expressed relative to Veh+Iono treatment). (**C**) Densitometry of (**Ci**) pro-IL-1α and (**Cii**) mature IL-1α protein levels from Figure 7Bii (expressed relative to Veh+Iono treatment). (**Ciii**) Cell lysates from Figure 7B were probed by western blotting for pro-IL-1α protein, and (**Civ**) densitometry of pro-IL-1α protein levels are shown (expressed relative to LPS Veh treatment). (**D**) Densitometry of (**Di**) pro-IL-1α and (**Dii**) mature IL-1α protein levels from Figure 7Ciii (expressed relative to Veh+Iono treatment). Data are presented as mean ± SEM. Data were analysed using repeated-measures one-way (A, Civ) or two-way (Bi, Bii, Ci, Cii) ANOVA with Dunnett’s post-hoc test (versus Veh treatment within each group). *P<0.05; **P<0.01; ***P<0.001.


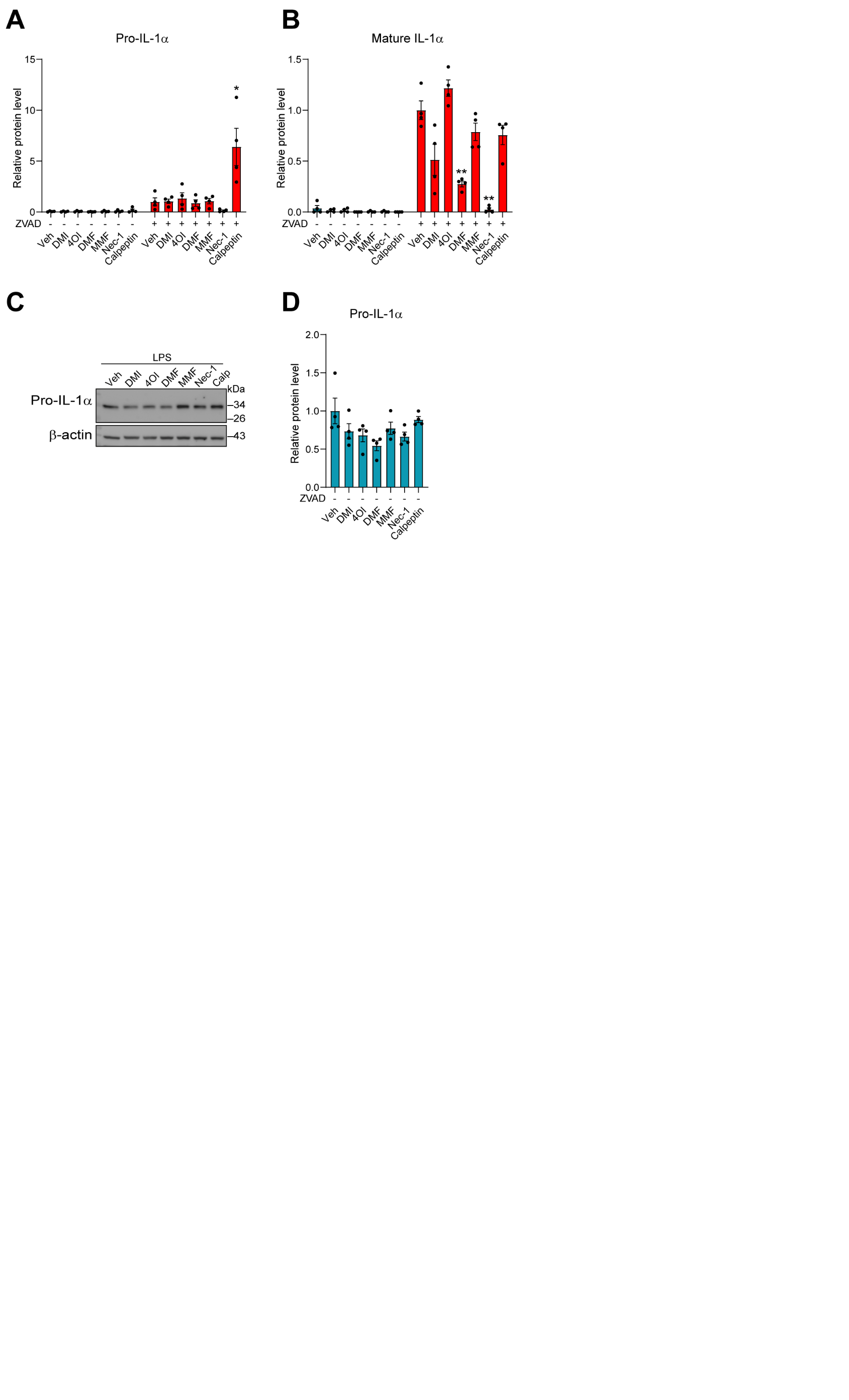


**Supplementary Figure 13. Densitometry analysis of western blotting from Figure 7Dii.** Densitometry of (**A**) pro-IL-1α and (**B**) mature IL-1α protein levels from Figure 7Dii (expressed relative to Veh+ZVAD treatment). (**C**) Cell lysates from Figure 7D were probed by western blotting for pro-IL-1α protein, and (**D**) densitometry of pro-IL-1α protein levels are shown (expressed relative to LPS Veh treatment). Data are presented as mean ± SEM. Data were analysed using repeated-measures one-way (D) or two-way (A, B) ANOVA with Dunnett’s post-hoc test (versus Veh treatment within each group). *P<0.05; **P<0.01.


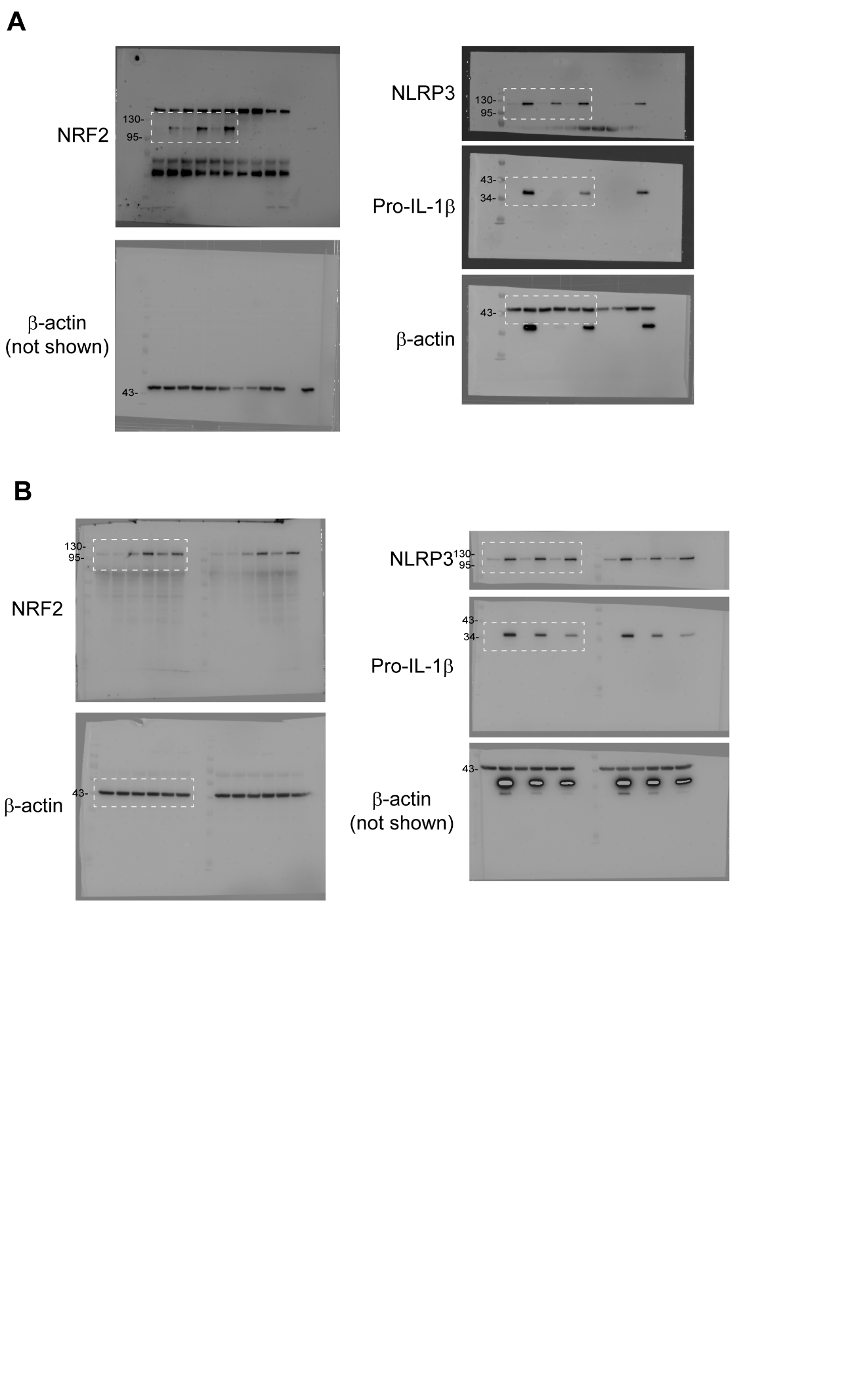


**Supplementary Figure 14.** **Uncropped western blots from (A) Figure 1A and (B) Figure 1C.**

**
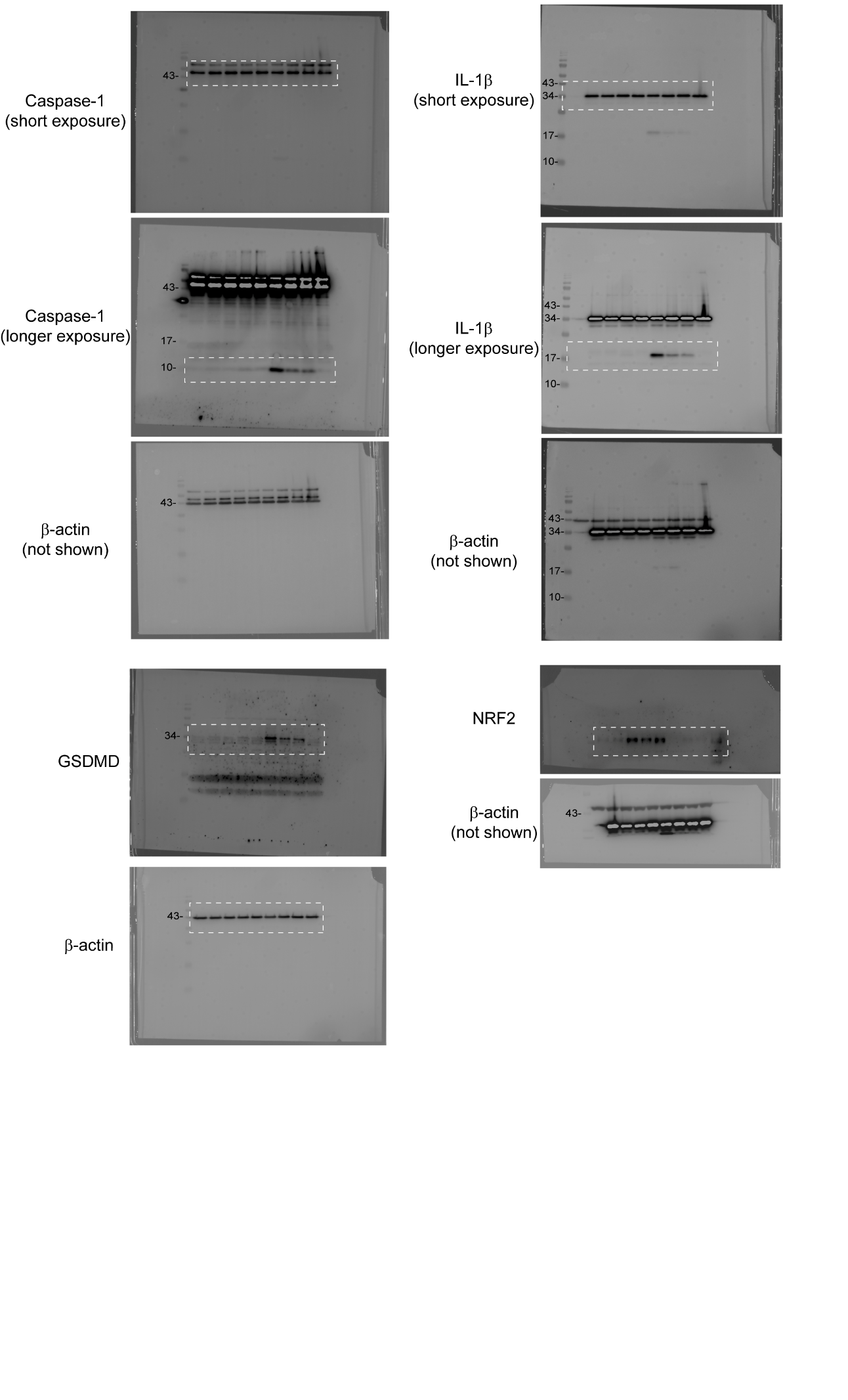
**

**Supplementary Figure 15.** **Uncropped western blots from Figure 2C.**

**
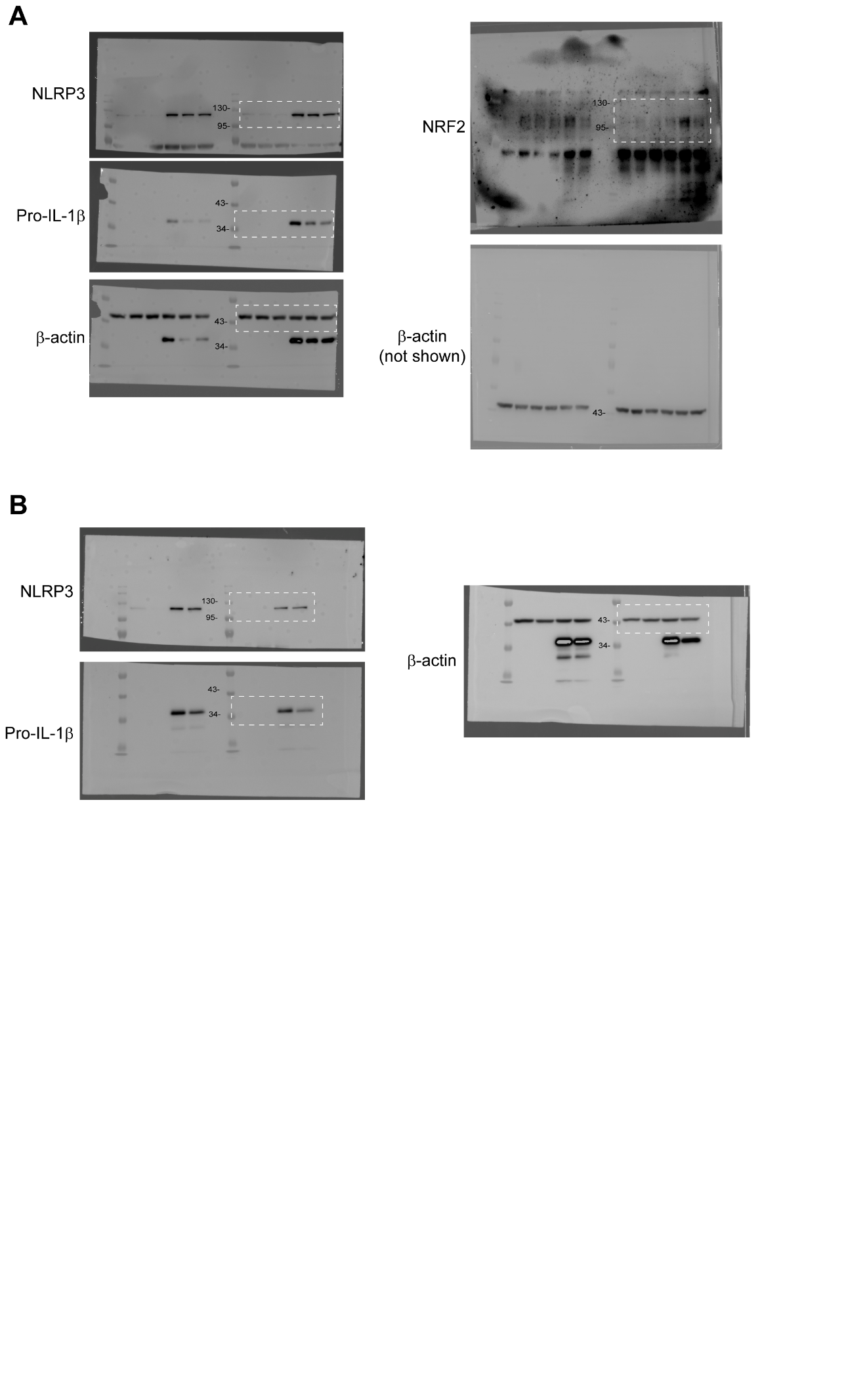
**

**Supplementary Figure 16.** **Uncropped western blots from (A) Figure 3Ai and (B) Figure 3Bi.**

**
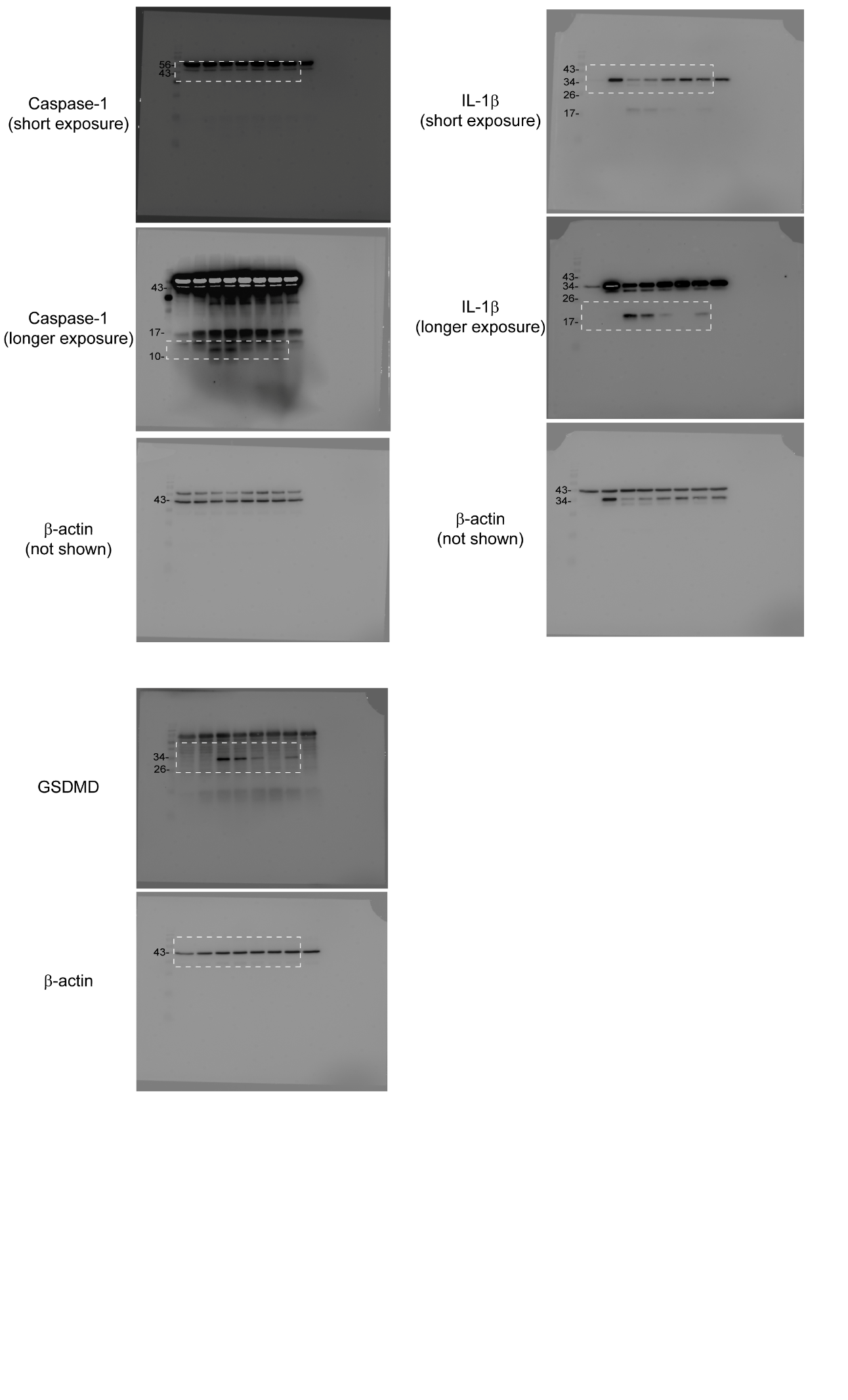
**

**Supplementary Figure 17.** **Uncropped western blots from Figure 4B.**

**
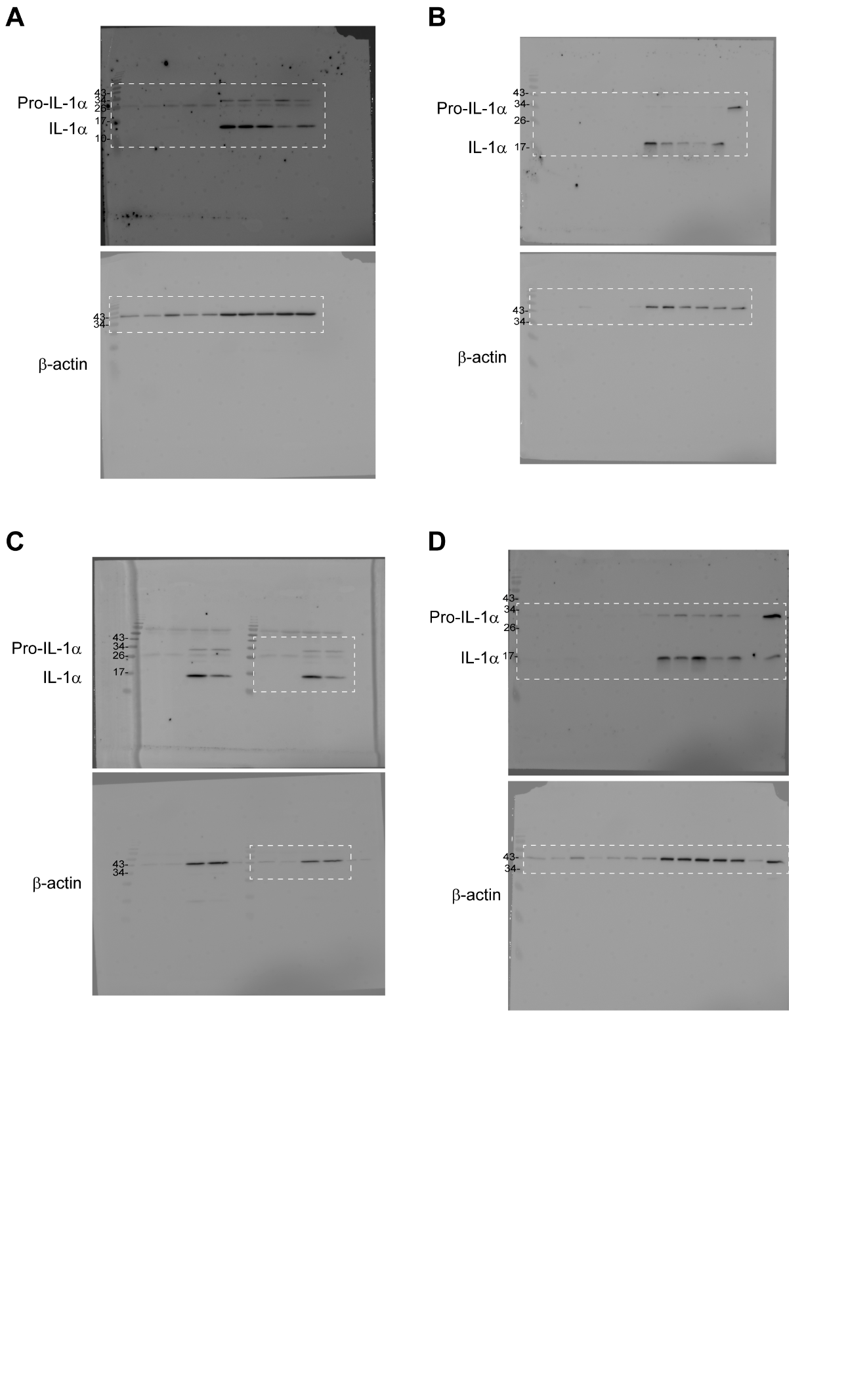
**

**Supplementary Figure 18.** **Uncropped western blots from (A) Figure 7Aii, (B) Figure 7Bii, (C) Figure 7Ciii and (D) Figure 7Dii.**


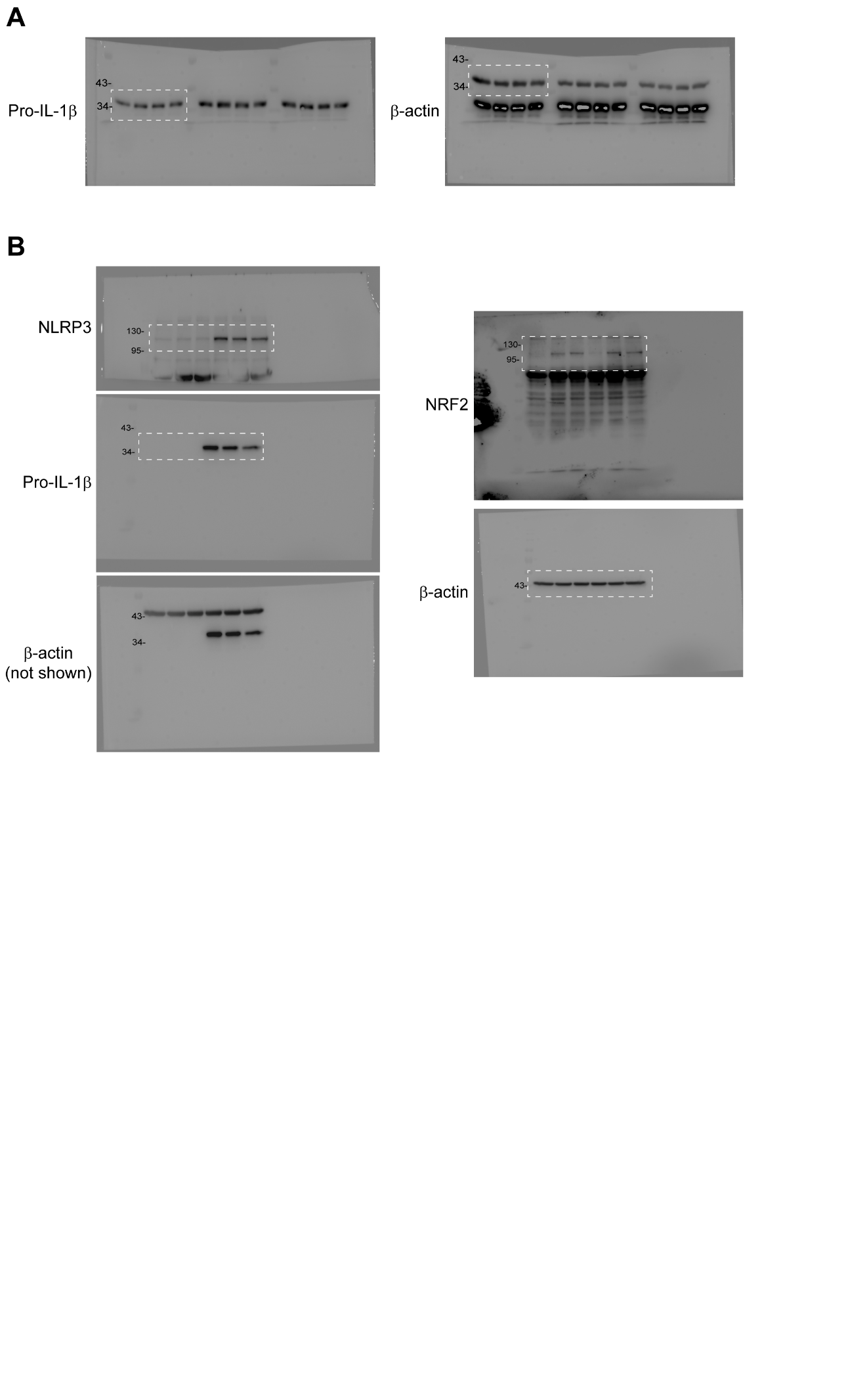


**Supplementary Figure 19.** **Uncropped western blots from (A) Supplementary Figure 3Ai and (B) Supplementary Figure 8Bi.**


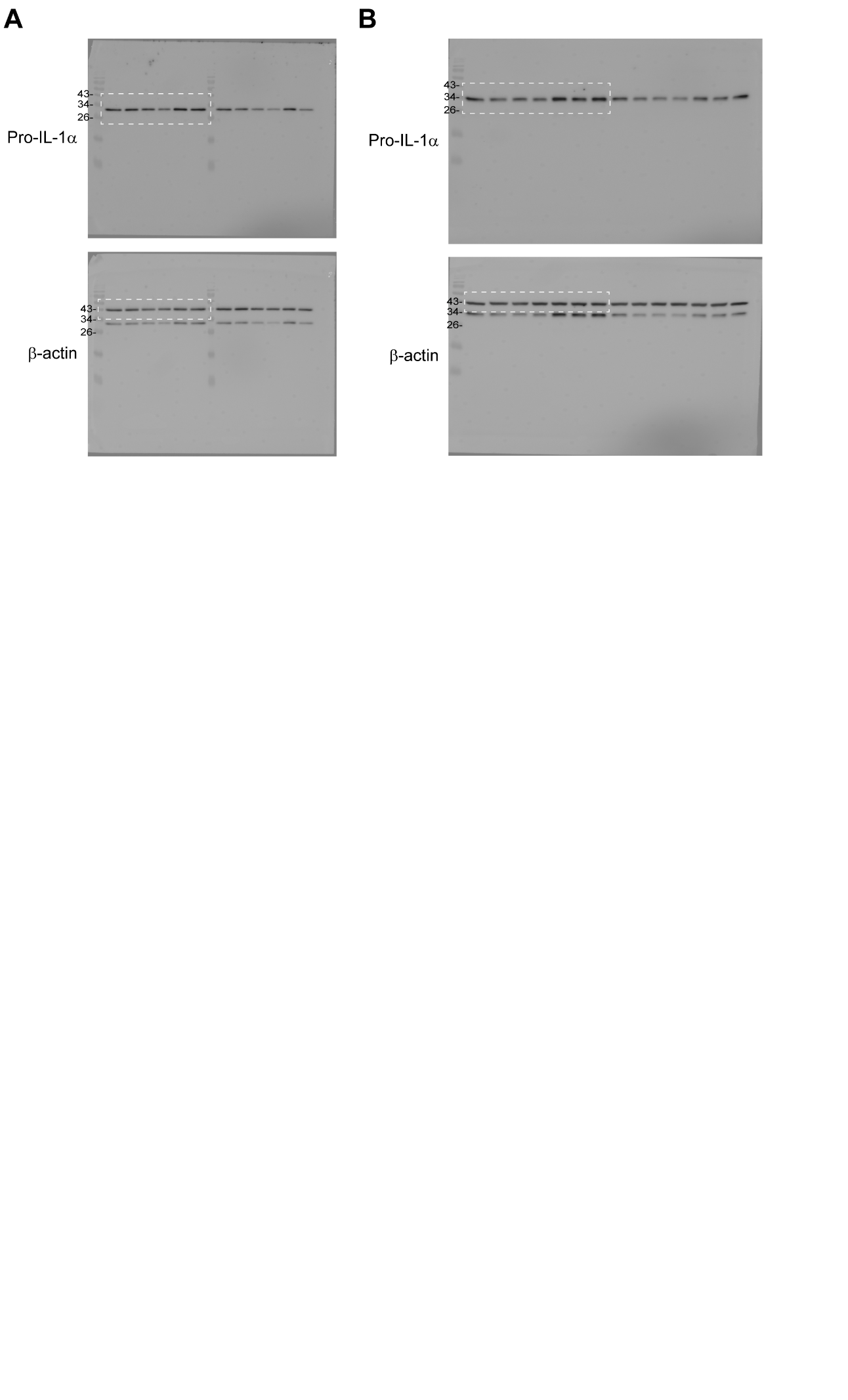


**Supplementary Figure 20.** **Uncropped western blots from (A) Supplementary Figure 12Ciii and (B) Supplementary Figure 13C.**
